# Glyphosate Primes Mammary Cells for Tumorigenesis by Reprogramming the Epigenome in a TET3-Dependent Manner

**DOI:** 10.3389/fgene.2019.00885

**Published:** 2019-09-27

**Authors:** Manon Duforestel, Arulraj Nadaradjane, Gwenola Bougras-Cartron, Joséphine Briand, Christophe Olivier, Jean-Sébastien Frenel, François M. Vallette, Sophie A. Lelièvre, Pierre-François Cartron

**Affiliations:** ^1^CRCINA, INSERM, Université de Nantes, Nantes, France; ^2^Equipe Apoptose et Progression tumorale, LaBCT, Institut de Cancérologie de l’Ouest, Saint Herblain, France; ^3^Cancéropole Grand-Ouest, réseau Epigénétique (RepiCGO), Nantes, France; ^4^LabEX IGO, Université de Nantes, Nantes, France; ^5^Service de toxicologie, Faculté de pharmacie de Nantes, Nantes, France; ^6^Department of Basic Medical Sciences, Purdue University, West Lafayette, IN, United States; ^7^Purdue University Center for Cancer Research, West Lafayette, IN, United States

**Keywords:** DNA methylation, ten-eleven translocation, breast cancer, hypomethylation, epigenetic mark

## Abstract

The acknowledgment that pollutants might influence the epigenome raises serious concerns regarding their long-term impact on the development of chronic diseases. The herbicide glyphosate has been scrutinized for an impact on cancer incidence, but reports demonstrate the difficulty of linking estimates of exposure and response analysis. An approach to better apprehend a potential risk impact for cancer is to follow a synergistic approach, as cancer rarely occurs in response to one risk factor. The known influence of glyphosate on estrogen-regulated pathway makes it a logical target of investigation in breast cancer research. We have used nonneoplastic MCF10A cells in a repeated glyphosate exposure pattern over 21 days. Glyphosate triggered a significant reduction in DNA methylation, as shown by the level of 5-methylcytosine DNA; however, in contrast to strong demethylating agent and cancer promoter UP peptide, glyphosate-treated cells did not lead to tumor development. Whereas UP acts through a DNMT1/PCNA/UHRF1 pathway, glyphosate triggered increased activity of ten-eleven translocation (TET)3. Combining glyphosate with enhanced expression of microRNA (miR) 182-5p associated with breast cancer induced tumor development in 50% of mice. Culture of primary cells from resected tumors revealed a luminal B (ER+/PR-/HER2-) phenotype in response to glyphosate-miR182-5p exposure with sensitivity to tamoxifen and invasive and migratory potentials. Tumor development could be prevented either by specifically inhibiting miR 182-5p or by treating glyphosate-miR 182-5p-cells with dimethyloxallyl glycine, an inhibitor of TET pathway. Looking for potential epigenetic marks of TET-mediated gene regulation under glyphosate exposure, we identified *MTRNR2L2* and *DUX4* genes, the hypomethylation of which was sustained even after stopping glyphosate exposure for 6 weeks. Our findings reveal that low pressure but sustained DNA hypomethylation occurring *via* the TET pathway primes cells for oncogenic response in the presence of another potential risk factor. These results warrant further investigation of glyphosate-mediated breast cancer risk.

## Introduction

Cancer results from interactions among genetic, epigenetic, environmental and lifestyle factors. Epigenetic modifications govern heritable changes in phenotypes regulated at the chromatin level without requiring DNA sequence alteration. They are strongly modulated by environmental and lifestyle factors. For instance, epigenetic differences between monozygotic twins have been shown to arise over their life-course (Fraga et al., 2005). In honeybees, fertile queens and sterile workers are alternative forms of the adult female that develop from genetically identical larvae following differential feeding with royal jelly. This specific nutrition is responsible for triggering modifications in the epigenome via a DNA MethylTransferase (DNMT) 3A-dependent mechanism (Kucharski et al., 2008) and histone post-translational modifications (Spannhoff et al., 2011). But, it is worrisome that certain exposures, as in farm environment, in early childhood appear to influence DNA methylation in genes related to asthma and allergy (Michel et al., 2013). Indeed, pollutants are powerful modulators of the epigenome. Over the past five years, 26 records related to the keywords “pollutant; epigenetic; cancer risk” can be found in the web of science (**Supplementary Figure S1**).

Especially, herbicides have been increasingly recognized as epigenetic modifiers. Exposure to Diuron was recently reported to affect the methylome of Pacific oysters (Rondon et al., 2017). In 2015, the International Agency for Research on Cancer (IARC) announced that the hazard of the herbicide glyphosate could be ranked as "probably carcinogenic to humans (Group 2A)”. Glyphosate was reported to induce the proliferation of human breast cancer cells via an impact on estrogen receptors (Thongprakaisang et al., 2013). This observation is supported by several other studies demonstrating that glyphosate can affect the activity of estrogen receptor alpha (ERα) and certain phenotypes of ERα positive cells within breast cancer cell populations (Mesnage et al., 2017; De Almeida et al., 2018; Sritana et al., 2018).

The impact of glyphosate on the distribution of methyl groups (or methylome) in the chromatin is extensive. Glyphosate exposure has been reported to induce 9,205 differentially methylated regions (DMRs) across the genome of Arabidopsis thaliana (Kim et al., 2017) and a decrease of DNA methylation in human peripheral blood mononuclear cells (Kwiatkowska et al., 2017).

Here, we present evidence that glyphosate induces global DNA hypomethylation (i.e. overall decrease of 5-methylCytosine (5mC) in the epigenome) in non-neoplastic mammary epithelial MCF10A cells and contributes to tumorigenesis in a “two-hit oncogenic model”. Our data also uncover a specific DNA hypomethylation signature of genes (i.e., local DNA hypomethylation) related to the TET3 pathway that might be used as epimark of glyphosate exposure.

## Results

### Exposure to Glyphosate Promotes TET3-Mediated Global DNA Hypomethylation in MCF10A Cells

**Figure 1:**
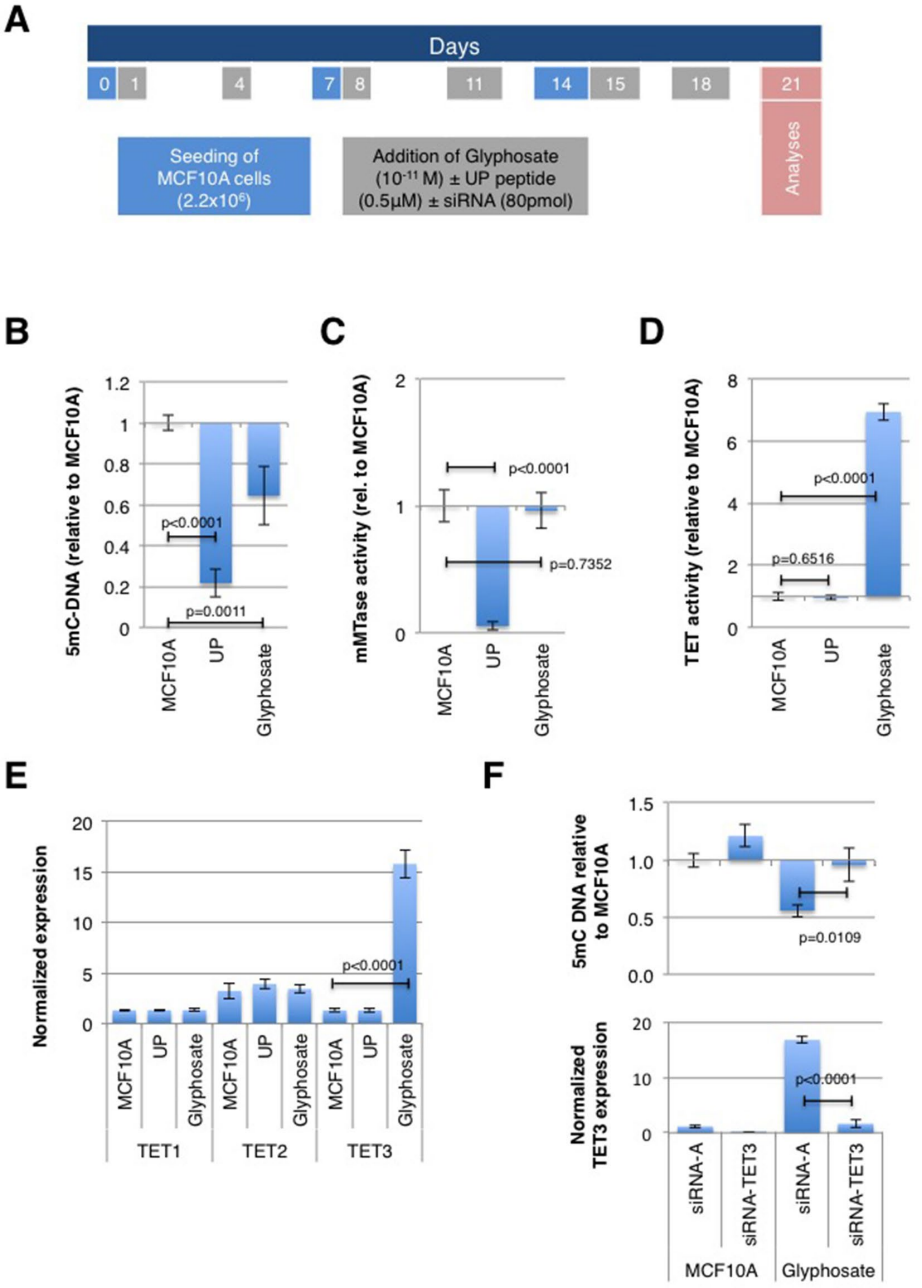
Glyphosate exposure promotes a TET3-mediated global DNA hypomethylation. MCF10A cells were treated according to a timetable shown in **(A)** and analyzed on day 21 of culture. (Explanations for color-coded days are located in corresponding color rectangles underneath the timeline. UP peptide promotes DNMT1/PCNA/UHRF1 disruption). **(B)** ELISA was used to measure the level of 5-methylcytosine (5-mC). **(C)** DMB assay was used to measure maintenance methyltransferase (mMTase). **(D)** TET assay. **(E)** In-Cell ELISA was used to quantify TET proteins. **(F)** MCF10A cells were transfected either with siRNA for TET3 or with control siRNA (siRNA-A) and treated with glyphosate (Glyphosate) or vehicle DMSO (MCF10A) according to a timetable shown in **(A)**. ELISA was used to measure the level of 5mC, and TET3 levels were determined by In-Cell ELISA and normalized to Janus Green staining intensity to account for differences in cell seeding density. For all assays, the bar graph displays the average ± standard deviation values of three independent experiments.

DNA hypomethylation has been shown to play a determining role in cancer development (Gaudet et al., 2003; Hervouet et al., 2010; Pacaud et al., 2014). To verify the impact of glyphosate exposure on the global level of DNA methylation, non-neoplastic breast epithelial MCF10A cells were treated with a low dose (10-11 M) of this herbicide every three to four days over 21 days, covering three passage numbers; whereas control cultures were treated with vehicle DMSO (**Figure 1A**). Several articles analyzing the effect of glyphosate on human cells have reported using 10-11 M (Thongprakaisang et al., 2013; Mesnage et al., 2017; Sritana et al., 2018). Indeed, 90% of MCF10A cells were viable as measured by XTT (2,3-bis-(2-methoxy-4-nitro-5-sulfophenyl)-2H-tetrazolium-5-carboxanilide) assay at this concentration (**Supplementary Figure S2**). Importantly, glyphosate 10-11 M is below the concentration detected in biological fluids (milk, serum, urine) (Yoshioka et al., 2011; Acquavella et al., 2004; Steinborn et al., 2016). As a control performed in parallel, MCF10A cells were exposed to carcinogenic UP peptide (0.5 μM) previously described to promote global DNA hypomethylation via the disruption of the DNMT1/PCNA/UHRF1 complex (Pacaud et al., 2014). As expected, there was a decrease in the level of 5mC-DNA in MCF10A cells treated with the UP peptide (**Figure 1B**). There was also a reduction in 5mC content in cells treated with glyphosate (**Figure 1B**), hence suggesting that glyphosate promotes a global DNA hypomethylation as per the definition given in the introduction.

The origin of glyphosate-mediated decrease in DNA methylation was assessed by measuring the levels of activity of maintenance methyltransferase (mMTase) and Ten-eleven translocation (TET), since a decrease of mMTase activity and an increase of TET activity are both causes of DNA hypomethylation. The mMTase activity remained unchanged in MCF10A cells treated with glyphosate (**Figure 1C**) while TET activity significantly increased in these cells (**Figure 1D**). Specifically, an ELISA-based assessment of the amount of the three TET family members, TET1, TET2 and TET3, revealed an overexpression of TET3 in MCF10A cells following exposure to glyphosate (**Figure 1E**).

To confirm that glyphosate promotes TET3-mediated global DNA hypomethylation in MCF10A cells, we analyzed the level of DNA methylation in MCF10A cells with siRNA-mediated TET3 down-regulation. ELISA results show that the presence of siRNA-TET3 abrogates TET3 overexpression and prevents DNA hypomethylation in cells exposed to glyphosate (**Figure 1F**).

### Glyphosate Exposure Is Tumorigenic for MCF10A Cells in a Two-Factor Hit Model

**Figure 2:**
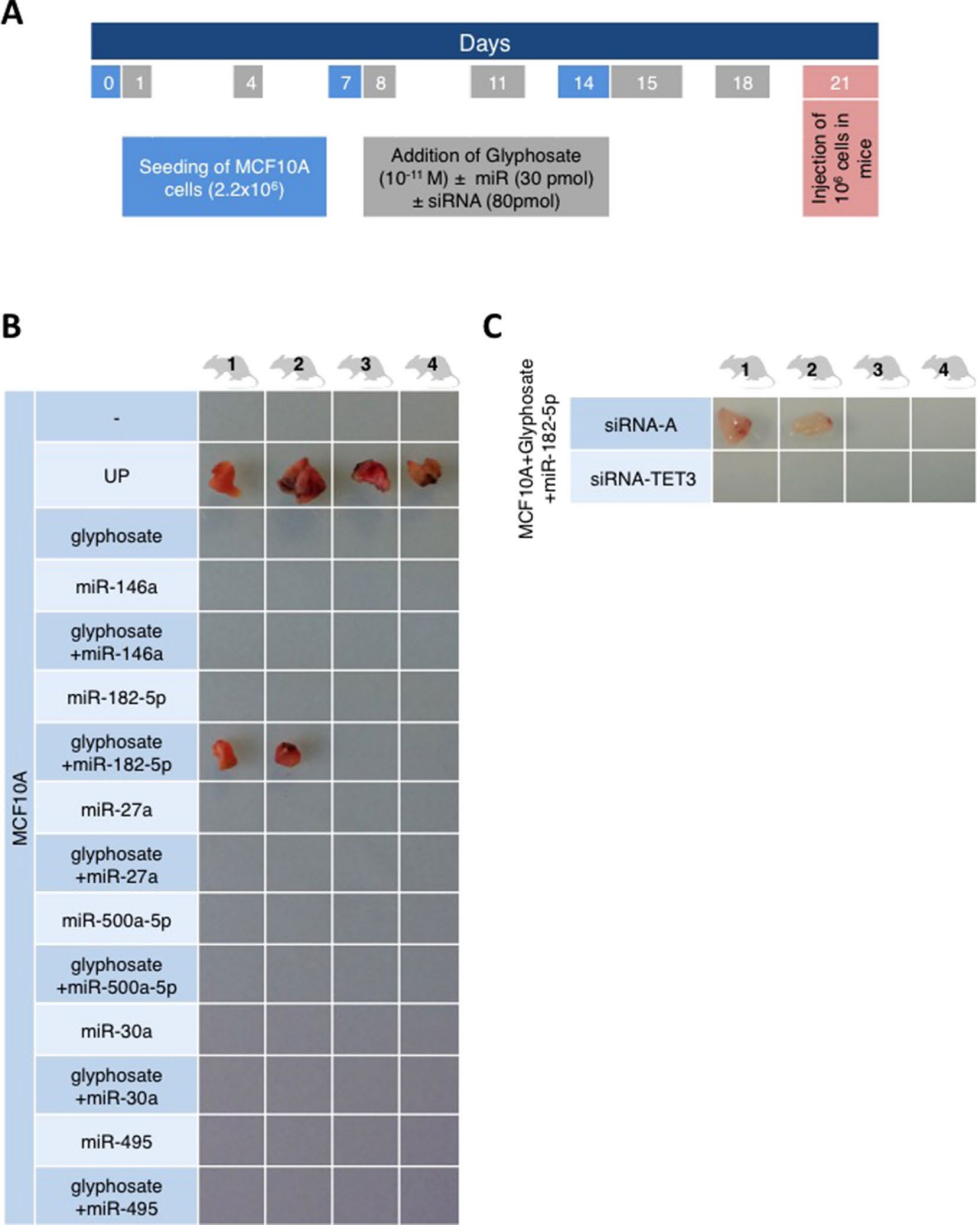
The combination of glyphosate exposure and miR-182 overexpression is tumorigenic for MCF10A cells in a two-factor hit model. **(A)** The timetable illustrates the experiment design. Explanations for color-coded days are located in corresponding color rectangles underneath the timeline. **(B** and **C)** Four mice were injected per condition. miRCury LNA miR mimics and siRNA for TET3 were used to overexpress miRs or siRNA in MCF10A cells. Mice were euthanized 21 days after the injection of cells, and the tumors were resected. The pictures show the resected tumors.

Global DNA hypomethylation is potentially tumorigenic (Gaudet et al., 2003; Hervouet et al., 2010; Pacaud et al., 2014). Therefore, MCF10A cells exposed to glyphosate were injected subcutaneously in Swiss nude mice. No tumors developed, whereas the control experiment with MCF10A cells exposed to the UP peptide led to visible tumor growth within 21 days in 100% of the mice (**Figure 2A**).

The Knudson’s hypothesis for cancer initiation suggests that several oncogenic hits cooperate to promote cancer. This hypothesis initially based on mutations can be transposed to risk factors beyond genetic alterations. Indeed, several microRNAs (miR) have been associated with cancer either as oncomiR (one hit) or suspected to promote cancer phenotype in light of their overexpression in cancers. To investigate the possibility of a two-factor hit oncogenic impact with glyphosate, six miRs associated with poor prognosis of breast cancer [miR-182-5p (Yu et al., 2017), miR-27a (Jiang et al., 2018), miR-500a-5p (Degli Esposti et al., 2017), miR-30a (di Gennaro et al., 2018), miR-495 (Cao et al., 2014), and miR-146a (Wang et al., 2016)] were transfected individually in MCF10A cells. For this purpose, miRs mimics were used, and their increased expression was confirmed by RTqPCR (**Supplementary Figure S3**). Tumor nodules were observed in two out of the four mice with subcutaneous injection of glyphosate-exposed MCF10A overexpressing miR-182-5p, whereas none of the other five miRs were associated with tumor formation (**Figure 2B**). Moreover, no tumor nodules were observed with subcutaneous injection of glyphosate/miR-182-5p/siRNA-TET3-exposed MCF10A, confirming that TET3 is implicated in glyphosate-mediated tumorigenic pathway (**Figure 2C**). The use of the Pan-cancer RNA-seq data available from the KM plotter database revealed that although TET3 overexpression is associated with a favorable overall survival in head and neck squamous cell carcinoma, thymoma, and thyroid carcinoma, it is associated with an unfavorable overall survival in breast cancer, as well as cervical squamous cell carcinoma, kidney renal papillary cell carcinoma, liver hepatocellular carcinoma, pheochromocytoma, paraganglioma, and uterine corpus endometrial carcinoma (**Supplementary File F1**).

**Figure 3:**
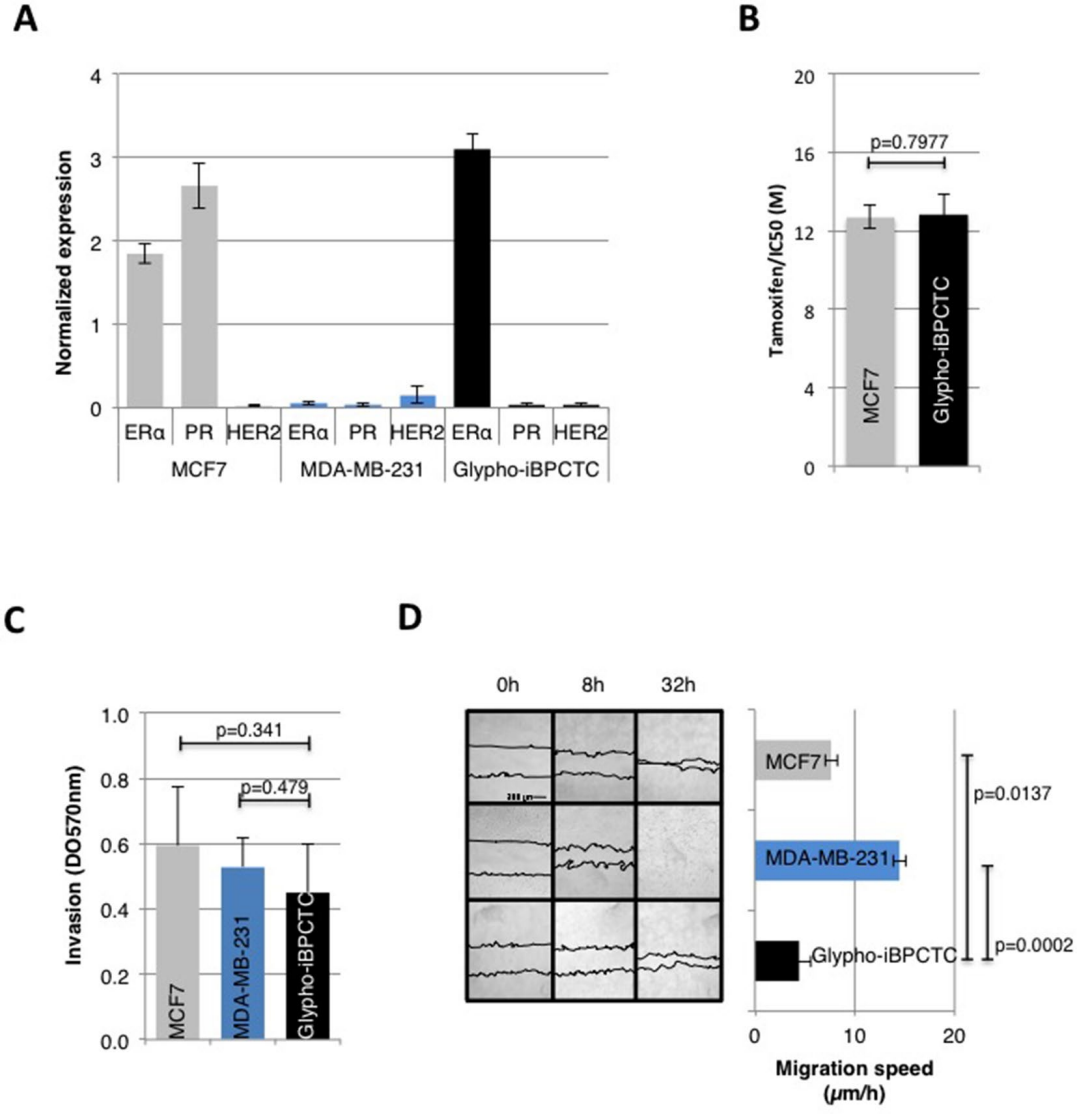
Primary cells from glyphosate-induced breast tumor display characteristics of malignant cells. **(A)** The expression levels of ERα, PR, and HER2 were estimated in MCF7 cells, MDA-MB-231 cells, and Glypho-iBPCTC primary cells using In-Cell ELISA. Normalization to Janus Green staining intensity was performed to account for differences in cell seeding density. The bar graph displays the average ± standard deviation values of three independent experiments. **(B)** Bar graph of the viability of MCF-7 and Glypho-iBPCTC cells treated with increasing doses of tamoxifen (0, 2, 4, 6, 8, 10, 16, 19, 22 μM). Viability was measured by an MTT test, and the results represent the average ± standard deviation values of six independent experiments. The IC50 for each cell type was calculated using the IC50 Calculator (ATT Bioquest). **(C)** Bar graph showing the invasion capacity of MCF-7, MDA-MB-231, and Glypho-iBPCTC cells measured by optical density (absorbance at 570 nm). n = 3. **(D)** Confluent cultures of MCF-7, MDA-MB-231, and Glypho-iBPCTC cells were subjected to the wound healing test. The average migration speed was obtained by calculating the ratio distance/time between each acquisition time. Left: Pictures were acquired immediately after seeding (0 h) and after 8 and 32 h of culture. The bar graph represents the average ± standard deviation values of three independent experiments.

We next compared several molecular signatures and phenotypic traits of primary cultures of tumor cells (PCTC) from glyphosate-induced breast tumors (Glypho-iBPCTC) with the ones of luminal A (MCF-7) and triple negative (MDA-MB-231) breast cancer cells. Only one of the two tumors led to viable Glypho-iBPCTC. In-cell ELISA confirmed that MCF7 and MDA-MB-231 cells were ERα+/PR+/HER2- (luminal A) and ERα-/PR-/HER2- (triple negative), respectively, and revealed that Glypho-iBPCTC were ERα+/PR-/HER2-, hence corresponding to a luminal B type of breast cancer with poorer outcome compared to ER+/PR+/HER2- subtype (Inic et al., 2014) (**Figure 3A**).

Tamoxifen/IC50 in MCF-7 and Glypho-iBPCTC were similar (**Figure 3B**). The QCM^™^ 24-Well Collagen-based cell invasion assay revealed that all cell strains had similar invasion capacity (**Figure 3C**), although scratch test indicated that Glypho-iBPCTC had the lowest migration ability compared to MCF-7 (p = 0.0137) and MDA-MB-231 cells (p = 0.0002) (**Figure 3D**). These results confirm that Glypho-iBPCTC display phenotypic traits associated with breast cancer cells *in vitro*.

### DMOG, a TET Inhibitor, Prevents Tumor Formation in Glyphosate-Challenged Cells

**Figure 4:**
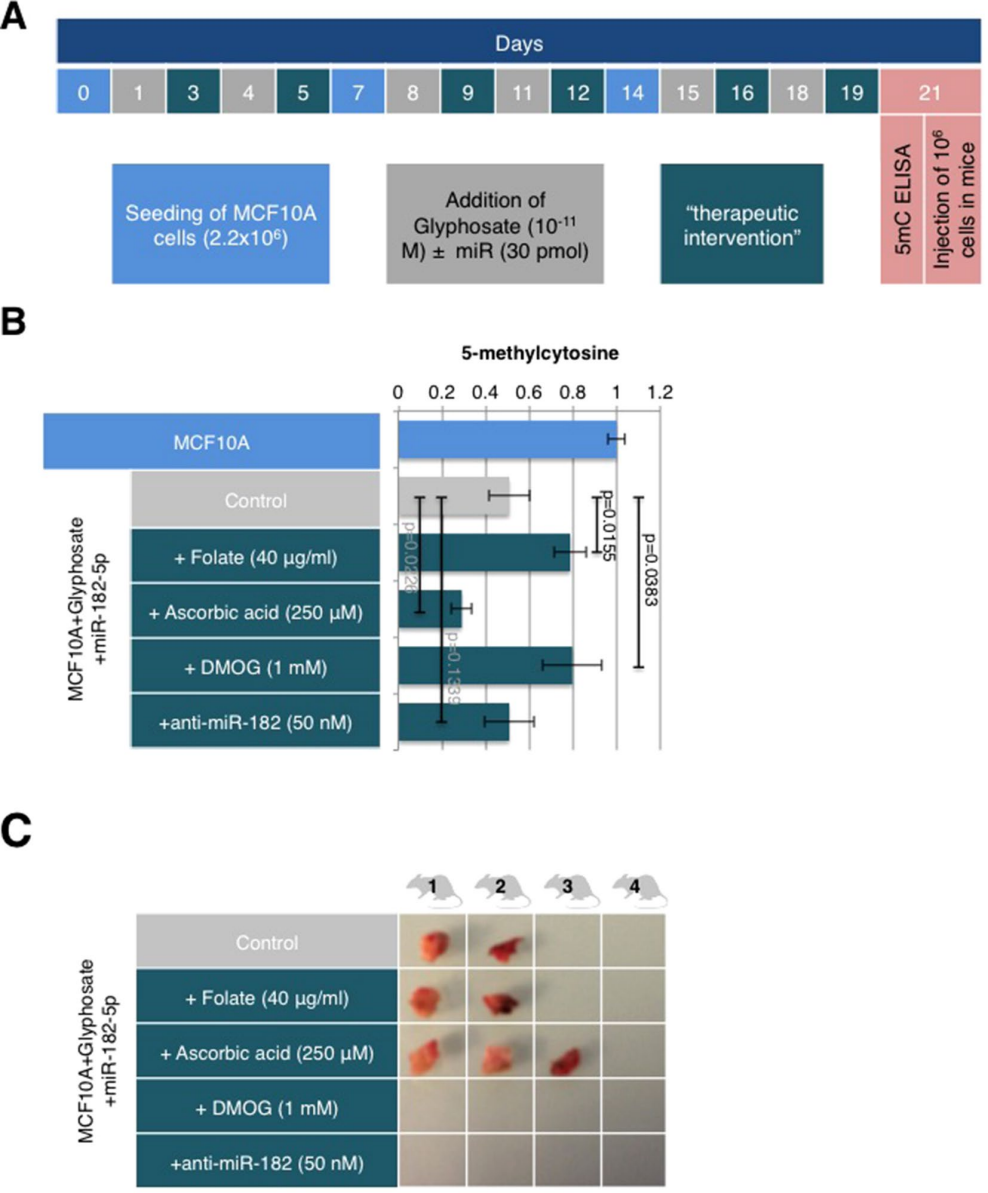
DMOG and anti-miR-182 prevent tumor onset but differentially impact 5-meC level. **(A)** The timetable illustrating the experiment design. Explanations for color-coded days are located in corresponding color rectangles underneath the timeline. Therapeutic interventions on MCF10A cells treated with glyphosate and miR as indicated were performed on days 3, 5, 9, 12, 16, and 19 with folate (40 μg/ml), ascorbic acid (250 μM), DMOG (1 mM), or anti-miR-182 (50 nM). **(B)** MCF10A cells were treated as shown in schedule A. DNA was extracted at day 21 and used in 5mC ELISA. The bar graph illustrates the levels of 5mC for the different conditions. **(C)** Mice were injected with the cells following the treatment schedule A and euthanized 21 days later. Shown are pictures of the resected tumors.

Some of the nutraceuticals/alicaments currently available target epigenetic pathways involved in normal homeostasis, notably those controlling DNA methylation. Like established epigenetic drugs, these sources of epigenetic modifiers offer great potentials to help determine the epigenetic path targeted by environmental factors and possibly revert the risk of tumorigenesis. MCF10A cells were transfected with miR-182-5p and exposed to 10^-11^ M of glyphosate (MCF10A^glyphosate/miR-182-5p^) every 3 to 4 days over a 21-day period. They were also simultaneously treated with 40 μg/ml folate, a promoter of DNA methylation (Hervouet et al., 2009; Cartron et al., 2012), or with 250 μM ascorbic acid, an activator of TET (Minor et al., 2013; Yin et al., 2013), 24 h after every glyphosate +/-miR treatment (**Figure 4A**). MCF10A^glyphosate/miR-182-5p^ cells were also treated in a similar manner with two therapeutic agents, an anti-miR-182-5p (50 nM) and dimethyloxallyl glycine (DMOG, 1 mM), a compound that blocks TET enzymatic activity (Zhang et al., 2017) (**Figure 4A**). For all of these conditions, we measured the global level of DNA methylation and tumor incidence compared to untreated MCF10A^glyphosate/miR-182-5p^ cells (control) at the end of the 21-day treatment sequence. As expected, folate and DMOG prevented glyphosate-induced DNA demethylation, whereas ascorbic acid further reduced DNA methylation in MCF10A^glyphosate/miR-182-5p^ cells, as shown by the level of 5mC (**Figure 4B**). Treatment with anti-miR-182-5p did not modify significantly the level of 5mC compared to control. Both folate and DMOG treatments were confirmed to indeed induce hypermethylation in several cell lines (**Supplementary Figure S4**). Of the two hypermethylating agents, DMOG and folate, only DMOG prevented tumor formation; there was no difference between folate and control treatments (50% of the mice displayed tumors). Ascorbic acid and glyphosate acting synergistically on DNA hypomethylation led to a 50% increase in tumor incidence. In contrast, although without an obvious impact on glyphosate-induced DNA hypomethylation, anti-miR-182-5p was able to prevent tumor formation (**Figure 4C**). These results confirm that both DNA demethylation and miR-182-5p are necessary for tumor onset. Importantly, the extent of DNA demethylation appears to set a threshold for tumor onset (i.e., the more hypomethylated, the higher the risk for tumor development).

### Glyphosate Exposure Induces Sustained TET3-Mediated Gene Demethylation

The hypomethylation induced by glyphosate treatment is sufficient for tumor onset when using a two-factor hit model with induced overexpression of miR-182-5p. Therefore, we investigated the possibility that an epimark of hypomethylation might be imprinted in the DNA.

**Figure 5:**
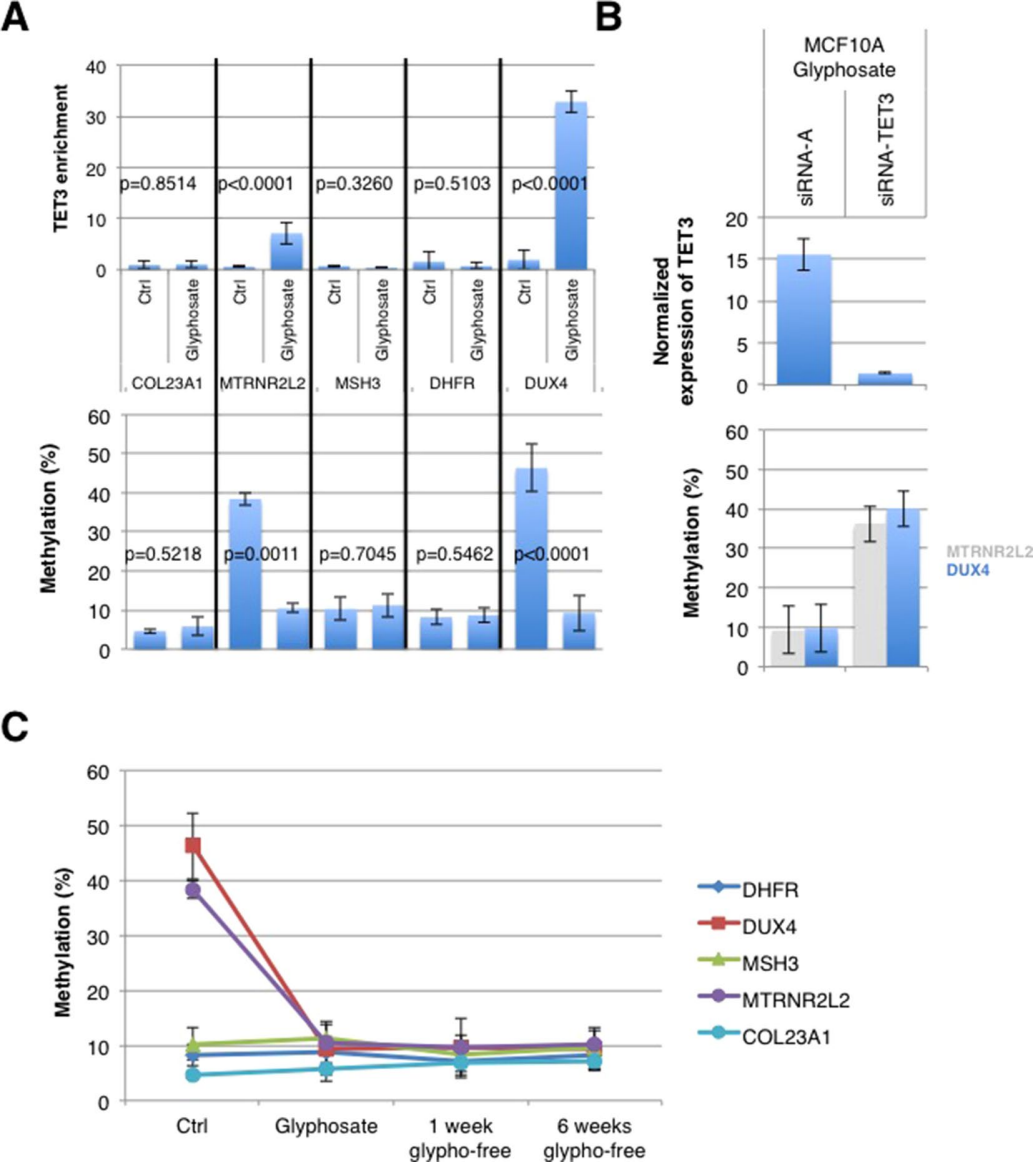
Glyphosate-induced TET3-mediated demethylation affects *MTRNR2L2* and *DUX4* genes. **(A)** MCF10A cells were treated with glyphosate for 21 days as in the schedule shown in **Figure 2**. The graphs illustrate TET3 enrichment (top) following chromatin immunoprecipitation (ChIP) and the methylation level measured by qMSRE (bottom) of five genes defined by the ChIP atlas as being TET3-targeted genes. **(B)** MCF10A cells were treated with glyphosate for 21 days (according to the timetable of **Figure 2**), with siRNA added concomitantly to glyphosate. Bar graph (top) of TET3 expression measured with In-Cell ELISA after treatment with siRNA-TET3 (sc94636) or control siRNA-A (sc94636). Normalization to Janus Green staining intensity was performed to account for differences in cell seeding density. Bar graph (bottom) of methylation levels of *DUX4* and *MTRNR2L2* genes as measured by qMSRE. **(C)** MCF10A cells were treated with glyphosate for 21 days (glyphosate) according to the schedule shown in **Figure 1** and then cultured in glyphosate-free medium for another 1 (1 week glypho-free) or 6 (6 weeks glypho-free) weeks. Shown is the graph of the methylation level of five TET3-dependent genes. “Ctrl” represents MCF10A cells without glyphosate exposure.

We postulated that the putative epimark induced by glyphosate might be the hypomethylation of TET3-targeted genes because TET3 mediates glyphosate-induced DNA hypomethylation. The chromatin immunoprecipitation (ChIP) atlas database identifies *MTRNR2L2*, *COL23A1*, *MSH3*, *DHFR*, and *DUX4* as the most frequently present in TET3-ChIP hits. According to this predictive finding, ChIP experiments using anti-TET3 antibody were performed for chromatin obtained from MCF10A cells treated or not with glyphosate for 21 days, such as described in **Figure 1A**. Interestingly, only *MTRNRL2* and *DUX4* genes were immunoprecipitated by TET3 in MCF10A cells treated with glyphosate. *COL23A1*, *MSH3*, and *DHFR* genes were not immunoprecipitated in both untreated and treated MCF10A cells. Thus, the prediction made by the ChIP atlas database was validated for *MTRNRL2* and *DUX4* genes and not for the *COL23A1*, *MSH3*, and *DHFR* genes, suggesting a context-dependent accessibility for this set of TET3-controled genes. Accordingly, quantitative methylation-sensitive restriction enzyme (qMSRE) revealed that *MTRNRL2* and *DUX4* genes were strongly methylated in control cells and became hypomethylated in MCF10A cells exposed to glyphosate (**Figure 5A**). The involvement of TET3 in the glyphosate-induced hypomethylation of *DUX4* and *MTRNR2L2* was confirmed by the abrogation with siRNA-TET3 of the glyphosate-induced hypomethylation of these genes (**Figure 5B**). Preliminary investigation of available breast tissue from breast cancer-free women confirmed the demethylation of *DUX4* and *MTRNR2L2* in a woman showing glyphosate exposure based on urinary test. However, the methylation status of the five genes immunoprecipitated by TET3, *MTRNR2L2*, *DUX4COL23A1*, *MSH3*, and *DHFR*, should be kept in consideration in the future because a woman with low glyphosate exposure displayed methylation on the five genes, hence suggesting that an epimark should consider the methylation status of all these genes in future investigations (**Supplementary Figure S5**).

The stability of epigenetic changes is an important factor for long-term risk determination. MCF10A cells were exposed to glyphosate for 21 days (as previously described; **Figure 1A**) and then cultured without glyphosate for 1 and 6 weeks. The *DUX4* and *MTRNRL2* hypomethylations remained stable, as shown by qMSRE, even after exposure to glyphosate has seized (**Figure 5C**). bc-GenExMiner and KM plotter indicated that a high expression of *DUX4* is associated with a poor prognosis, suggesting that genes controlled by TET3 might deserve additional scrutiny in breast cancer pathogenesis (**Supplementary File F2**).

## Discussion

The impact of glyphosate on human health has been analyzed and discussed for several years now (Gillezeau et al., 2019). Recently, glyphosate exposure was correlated with shortened gestational lengths (Parvez et al., 2018), and the level of glyphosate excretion was associated with steatohepatitis and advanced liver fibrosis in patients with fatty liver disease (Mills et al., 2019). However, the multiple research studies that investigated the tumorigenic effect of glyphosate as the sole risk factor had not led to convincing evidence of its implication.

**Figure 6:**
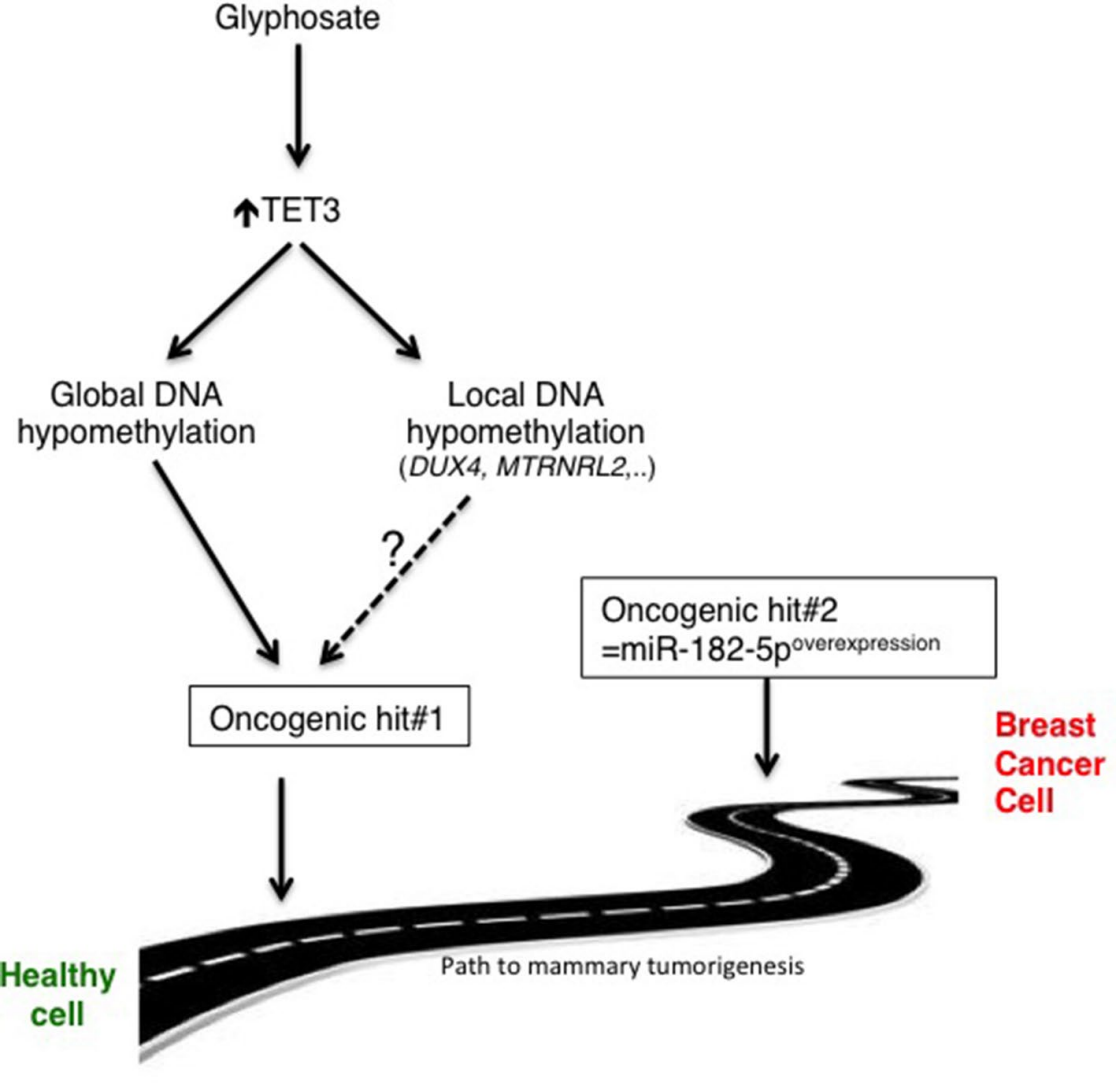
Schematic representation of the proposed glyphosate-induced mammary tumorigenesis path. Whether *DUX4* and *MTRNRL2* are involved in oncogenesis remains to be determined.

It is assumed that only 5–10% of cancers are directly caused by inherited genetic abnormalities. The remaining 90% of cancers are linked to environmental factors that directly or indirectly affect DNA, possibly triggering genetic defects or aberrations in the reading and/or expression of DNA (Perera, 1997; Anand et al., 2008). Environmental and lifestyle factors are pleiotropic and include diet, tobacco, infections, obesity, alcohol, radiation, stress, physical activity, exposure to heavy metals and other pollutants, such as glyphosate. We are reporting that glyphosate exposure is not oncogenic by itself, but it acts as an oncogenic hit factor that, combined with another oncogenic hit, promotes the development of mammary tumors. At the molecular level, our findings demonstrate that glyphosate exposure can predispose breast cells to tumorigenesis *via* epigenetic reprogramming occurring *via* TET3-mediated global and local DNA hypomethylation (**Figure 6**).

We and others have identified that global DNA hypomethylation promoting tumorigenesis may be caused by a deficiency of the DNMT1/PCNA/UHRF1 complex or of DNMT1 expression as shown in astrocytes, pulmonary fibroblasts, mesothelial cells, and breast cells (Gaudet et al., 2003; Hervouet et al., 2010; Pacaud et al., 2014). We show that glyphosate-mediated DNA hypomethylation is associated with TET3 overexpression instead of the DNMT1 pathway. The lower degree of DNA hypomethylation reached *via* the glyphosate-TET3 path compared to that reached *via* UP peptide-DNMT1 path that is capable of inducing tumor onset suggests that a great intensity of global DNA hypomethylation could act as an oncogenic event, while a moderate intensity of global DNA hypomethylation might be considered a predisposing factor to cancer. The fact that active DNA demethylation orchestrated by TET can occur in resting (nondividing) cells representing the majority of breast cells (in contrast to DNMT activity that requires cell proliferation) confers to TET-mediated mechanism a potentially higher degree of danger for cancer development.

The implication of TET proteins in breast cancer growth and metastasis has been strongly documented (Sun et al., 2013; Yang et al., 2015), and the level of hypomethylation of triple-negative breast cancer has been associated with TET1 DNA demethylase activity (Good et al., 2018). In the latter article, it is proposed but not shown that TET1 might act as an oncogene by leading to aberrant hypomethylation. Our findings demonstrate that the hypothesis of an involvement of TET-mediated DNA hypomethylation in cancer onset was correct. Notably, siRNA-TET3 abolished the presence of glyphosate-induced global and local *DUX4* and *MTRNR2L2* hypomethylation, as well as tumorigenesis. Our data feed the ongoing debate regarding whether TET3 exerts an oncogenic role or a tumor suppressor role. For the latter role, TET3 might act by inhibiting epithelial-to-mesenchymal transition in ovarian and melanoma cancers (Ye et al., 2016; Gong et al., 2017). But our analysis with KM plotter database revealed a potentially unfavorable outcome for breast cancers when TET3 is overexpressed (**Supplementary File F1**).

Our work shows that two epigenetic events (global DNA hypomethylation and overexpression of a miR) cooperate to promote breast cancer. Other epigenetic events described to be involved in breast cancer development include the reduction of H3K9 acetylation *via* TIP60 downregulation that promotes ER-negative tumors (Bassi et al., 2017; Judes et al., 2018). Histone acetyltransferase p300 activity and BIM1-mediated histone H2A ubiquitination that remodel chromatin are also two epigenetic events described as promoters for the development of aggressive breast tumors. A body of literature reports that miRs also play a crucial role in mammary tumorigenesis. In addition to oncogenic miRs, there are also miRs acting as tumor suppressors. For example, loss of miR-10b delays oncogene-induced mammary tumorigenesis (Kim et al., 2016), and overexpression of miR-489 inhibits HER2/neu-induced mammary tumorigenesis (Patel et al., 2019). Since the expression of miR depends on epigenetic control, it seems that either an extensive global hypomethylation of DNA (like with UP peptide) or a less extensive global hypomethylation associated with local epigenetic alterations affecting a miR might lead to tumor onset. The mechanisms associated with specific targeting of miR expression remain to be understood.

Breast cancer susceptibility has been statistically linked to epigenetic age acceleration and CpG island methylation (Ambatipudi et al., 2017). An important question is whether exposure to pollutants that are detrimental to epigenetic homeostasis might replace or synergize with age-related epigenetic changes and thus lead to the increase in earlier onset of breast cancer that is now documented. This possibility is further supported by our preliminary observation that the luminal B subtype of tumor (ER+/PR-/HER2-) triggered by glyphosate exposure combined with miR-182-5p overexpression is associated with poorer outcomes than the frequent ER+/PR+/HER2-luminal A type of tumor. Indeed, luminal B type of tumors have been found to be most common in young patients (Goksu et al., 2014). This phenotype obtained from one tumor produced in mice will have to be confirmed with additional means; in any case, epigenetic markers of risk would be a prime resource to help curve the incidence. There exist already DNA methylation markers that add to the prediction of tertiary and secondary outcomes over and beyond standard clinical measures (Terry et al., 2016).

In the MCF10A model, glyphosate-induced DNA hypomethylation can be detected *via* the methylation level of only two of the five genes predicted to be controlled by TET3, *MTRNR2L2* and *DUX4* genes. Even if several other factors than glyphosate-induced TET3-mediated DNA hypomethylation (such as chromatin structure, other epimark, etc.) can govern the methylation status of the five genes, *MTRNR2L2, DUX4, COL23A1, MSH3*, and *DHFR*, our preliminary data with human samples support the idea that the study of the methylation status of these five genes might be important to obtain a marker of risk based on a MethylGlypho score. We are now pursuing this direction of research by detecting and analyzing this 5-gene TET3-dependent epimark in blood samples. Possibly, glyphosate-induced methylome reprogramming might be used for the detection of an increased risk for breast cancer in women living in an environment conducive to this type of pollution.

Due to their concomitant expression during tumorigenesis associated with glyphosate-induced DNA hypomethylation, *DUX4* and *MTRNR2L2* may appear as players in this process instead of only be considered potential biomarkers. Results with KM plotter and bc-GenExMiner indicate that *DUX4* level is negatively associated with breast cancer prognosis. No data seems available on *MTRN2L2* in these databases. Based on the literature, *DUX4* could act as an oncogene in various sarcomas and hematological malignancies (Dib et al., 2019), while we could not find information in the literature revealing a putative oncogenic role for *MTRNR2L2*. These TET3-controlled genes are worth further investigation to establish their causal effect in mammary tumorigenesis in future work.

Knowing the epigenetic pathway involved in glyphosate-mediated risk increase might lead to prevention strategies to follow detection of the epigenetic risk. Our findings suggest that TET-specific inhibitor DMOG might be a plausible therapeutic intervention since it gave a satisfactory response on both DNA methylation and tumor incidence. It would act by limiting TET3-mediated global DNA hypomethylation. In contrast, global remethylation of DNA by folate that has been considered for possible preventive effect is insufficient to prevent tumor incidence in the case of glyphosate exposure (Hervouet et al., 2009; Cartron et al., 2012). Another interesting direction would be to limit the intake of ascorbic acid since it not only further reduced DNA methylation but also increased tumor incidence in mice. The epigenetic pathway leading to DNA hypomethylation is an important aspect to consider for further translational work on breast cancer risk.

## Materials and Methods

### Cell Culture and Transfection

MCF10A cells were cultured in DMEM/F12 supplemented with 5% horse serum (Invitrogen, Cergy Pontoise, France), 500 ng/ml hydrocortisone (Sigma-Aldrich, France), 100 ng/ml cholera toxin (Sigma-Aldrich, France), 10 μg/ml insulin (ThermoFisher, France) and 20 ng/ml epidermal growth factor (EGF, Sigma-Aldrich, France), penicillin (100 U/ml), and 2 mmol/L L-glutamine. MCF7 and MDA-MB-231 cells were cultured in DMEM medium (Invitrogen) all supplemented with 5% FCS and 2 mM l-glutamine. Glyphosate (CAS 1071-83-6, sc-211568) was purchased from Santa-Cruz (France), and a 10^-8^-M stock solution was prepared in DMSO every week. Glyphosate was diluted directly in fresh cell culture medium and was fed to the cells at the time points indicated in the results section.

For the transfection of RNAs, we used miRCury LNA miR mimics for the has-miR-146a, has-miR-182-5p, has-miR-27a, has-miR-500a-5p, has-miR-30a, and has-miR-495 (Qiagen, France), siRNA for siRNA-T ET3 (sc94636) and control siRNA-A (sc94636) and HIPerfect Transfection Reagent (Qiagen, France). All miRs showed similar transfection efficiency (10- to 15-fold change, as measured by RTqPCR) (**Supplementary Figure S3**).

### DNA Extraction, 5mC ELISA, and qMSRE

A QIAcube automate and QIAmp DNA Mini QiaCube kit (Qiagen, France) were used to isolate DNA.

The quantification of 5mC was performed using the 5mC DNA ELISA Kit (Zymo Research-Euromodex, France) according to the manufacturer’s instructions. The 5mC DNA ELISA Kit estimates the number of 5mC on DNA without distinction of localization; therefore, we used the term of global DNA methylation level when referring to results obtained *via* this mode of quantification.

Next, DNA methylation was quantified by qMSRE. Digestions were performed with adequate restriction enzymes, HpaII and AciI (NEB, France). Typically, 1 ng of genomic DNA was digested with 40 U of enzymes at 37°C for 2 h in 50 μl of reaction. Control samples were treated in the same way but without addition of the enzyme. Five microliters of digestion mixture were used for qPCR. The QuantiFast SYBR Green PCR Kit and Rotor-Gene Q (Qiagen, France) were used to perform the qPCR. Primers were MSH3: TTTCTCCAGGGCTGGGACTTTG and CCCGACTGGATTCCCCTTTTCT; DHFR: AAACCTCAGCGCTTCACCCAAT and TGATAGGGCTGGAGGAGGAAG; DUX4: CGACACCCTCGGACAGCA and TCAAAGCAGGCTCGCAG; COL23A1: TCTCCAGGCCAGAAACAGTCTT and ATTTAGAGAGGCAGGGTCGAGA; and MTRNR2L2: ACCCCACCTGTTTACCAA and GCTACCTTTGCACGGTTAGGG.

### Tumor Xenografts in Nude Mice

Cells were harvested by trypsinization, washed and resuspended in saline buffer. Cell suspensions were injected subcutaneously into the flank of 7 to 8-week-old mice (Janvier, France) in 100 μl of sterile PBS. Tumor volume based on caliper measurements was calculated using the modified ellipsoidal formula [Tumor volume = 1/2 (*length* × *width*^2^)] according to previously published work (Cartron et al., 2012). At the end of the observation period, the mice with xenograft tumors were euthanized, and the tumor tissues were removed for analysis.

The experimental procedures with animals were in accordance with the guidelines of Institutional Animal Care and the French National Committee of Ethics. In addition, all experiments were conducted according to the Regulations for Animal Experimentation at the *Plateforme Animalerie* in the *Institut de Recherche en Santé de l’Université de Nantes* (IRS-UN) and approved by the French National Committee of Ethics. The number of mice was restricted to four per condition to limit the number of animals to the necessary minimum as in previous studies (Hervouet et al., 2010; Pacaud et al., 2014) based on the fact that we anticipated to detect a highly frequent tumorigenic event (frequency superior to one to four for tumorigenesis).

### Establishment of Tumor Cells From Xenografts (PCTCdX)

PCTCdX (here named Glypho-iBPCTC) were obtained after mechanical dissociation. Briefly, resected tumor tissue from mice was cut into pieces of 1–5 mm^3^ and plated in a 60-mm^2^ tissue culture dish with DMEM containing 10% FBS and antibiotics. Minced pieces of tumor were incubated with 200 U/ml collagenase I (Sigma) and 500 U/ml DNaseI (Sigma) in PBS for 1 h at 37°C with vigorous constant agitation. The single-cell suspension was filtered through a 70-mm cell strainer (BD Falcon), washed with PBS, and then placed in DMEM-10% FBS. Cell cultures were split 1:5 when confluent.

### Migration Assay

Cells (3 × 10^5^) were seeded in six-well plates, cultured until they reached 80–90% confluence, and treated with 10 μg/ml of mitomycin C (Sigma, France) for 2 h (to prevent cell proliferation). The monolayer of cells was scratched using a two-well silicone insert (Ibidi, Germany). Cell migration was monitored by microscopy (Incellis Cell Imager, Bertin, France). The images acquired at different time points (0, 4, 8, 24, 28, 32, and 48 h) for each sample were analyzed quantitatively. For each image, distances between one side of the wound and the other side were measured with ImageJ software; the mean value of 10 measurements all along the wound was recorded. The average migration speed was obtained by calculating the ratio distance/time along the time course.

### Invasion Assay

All of the procedures were performed according to the manufacturer’s instructions (QCM 24-Well Collagen-Based Cell Invasion Assay, Millipore, France). In brief, 200 μl of serum-free medium containing 2 × 10^5^ cells were added into the invasion chamber, with the bottom well of the 24-well plate containing 500 μl of complete medium. After 72 h of incubation at 37°C, the medium was removed, and the cells were stained by placing the chamber in staining solution for 20 min at room temperature. Cells that did not invade were carefully removed from the top side of the chamber using a cotton swab. The stained chamber was inserted into a clean well containing 200 μl of extraction buffer for 15 min at room temperature. A total of 100 μl of extracted (stained) solution from the chamber was transferred into a 96-well plate, and the optical density was measured 570 nm using a spectrophotometer.

### Viability Assay: MTT and XTT Tests

A cell suspension containing 10^5^ cells was prepared, and 100 μl was distributed in sixplicates in a 96-well plate. After 24 h of incubation at 37°C and 5% CO_2_, cells were exposed to tamoxifen for 48 h. Tamoxifen was first diluted 10 times in dimethyl sulfoxide (DMSO) and then further diluted in DMEM containing 4.5 g/L glucose, 1% SVF, 1% glutamine, 1% penicillin-streptomycin at the desired concentrations. Following treatment, 10 μl of MTT (10 μg/ml) (VWR Chemicals, France) was added in each well, and the cells were incubated for 3 h. Finally, the medium containing MTT was removed, and 200 μl/well of DMSO was added to measure the optical density at 570 nm using a spectrophotometer.

For the XTT test, we used the XTT Assay Kit (ab232856, Abcam, France) according to the manufacturer’s instructions. Briefly, 10^5^ cells were seeded in 100 μl of culture medium in each well of a 96-well plate. After 24 h of incubation at 37°C and 5% CO_2_, cells were treated with adequate drugs. Then, 10 μl/well of XTT mixture was added for an incubation of 2 h at 37°C and 5% CO_2_. Finally, absorbance was measured at 450 nm.

### Breast Tissue and Urine Samples

Human samples were collected from the Réseau des tumorothèques du Cancéropole Grand-Ouest and Institut de Cancérologie de l’Ouest (ICO, http://www.ico-cancer.fr).

In accordance with regulations, all subjects signed a specific informed consent form for this biocollection approved by an Ethics Committee (CPP OUEST IV, n°18/16), the French State Department for National Education, Higher Education and Research (Ministère de l’Education Nationale, de l’Enseignement Supérieur et de la Recherche, N° DC-2015-2457) and the Commission Nationale de l’Informatique et des Libertés (CNIL) (compliance commitment to MR 001). The glyphosate concentration in urine samples was obtained using Glyphosate kit (Novakits, France).

### mMTase and TET Activities

TET activity was determined using the Epigenase 5mC-Hydroxylase TET Activity/Inhibition Assay Kit (Colorimetric; Epigentek/Euromedex, France) according to the manufacturer’s instructions. Dnmts-magnetic beads (DMB) assays were performed to estimate mMTase, such as initially described (Yokochi and Robertson, 2002). Briefly, a typical methylation reaction required 50 µg of nuclear extract (Nuclear extract kit, Active Motif, France), 125 nM DNA oligonucleotides (probes), and 900 nM tritium-labeled AdoMet (1 mCi/ml; #NET155V001MC; PerkinElmer, France) in reaction buffer (50 mM Tris, pH 8.0, 5 mM EDTA, 10% glycerol, 0.5 mM phenylmethylsulfonyl fluoride). After incubation at 37°C for 1 h, reactions were quenched with an equal volume of magnetic beads suspension and incubated for 15 min at room temperature. Next, the beads were magnetically isolated from the reaction mix, and tritium incorporation was measured by scintillation counting.

### In-Cell ELISA

In-cell ELISA was performed using the In-Cell ELISA Kit (Abcam, France) according to the manufacturer’s instructions and after a fixation step performed with 4% of paraformaldehyde solution (10 min at room temperature). Primary antibodies were incubated overnight at 4°C. Adequate HRP-conjugated secondary antibodies were incubated for 1 h at room temperature. Detection was performed at 450 nm.

After the washes, cells in each well were incubated with 1X Janus Green Stain for 5 min at room temperature, according to the manufacturer’s instructions. Data were expressed in normalized unit, according to the following calculation: (HRPsignal ‘minus’ HRPsignal in absence of primary antibody)/(Janus Green signal ‘minus’ Janus Green signal in absence of cells).

Antibodies used were anti-TET1 (sc163446, Santa Cruz, France), anti-TET2 (sc398535, Santa Cruz), anti-TET3 (sc139186, Santa Cruz), anti-ERα (sc8002, Santa Cruz), anti-PR (sc130071, Santa Cruz), and anti-HER2 (sc-393712, Santa Cruz).

### ChIP Analyses

ChIP was performed using the ChIP-IT Express kit (Active Motif, France) according to the manufacturer’s instructions. The cross-linking step was performed by treating the cells with 37% formaldehyde solution for 15 min at room temperature. Sonication was performed with the Bioruptor Plus (eight cycles 30 s on/90 s off) (Diagenode, France). The QuantiFast SYBR Green PCR Kit and Rotor-Gene Q (Qiagen, France) were used to perform the qPCR. Antibodies used were Anti-IgG (Abcam, AB2410) and anti-TET3 (sc139186, Santa Cruz).

### Statistical Analysis

All experiments were done at least in biological triplicates. Differences in means were assessed using Student t test, and the degree of correlation between two parameters was calculated using Pearson’s test. *P* < 0.05 was considered significant.

## Data Availability

All datasets generated for this study are included in the manuscript and the **Supplementary Files**.

## Ethics Statement

The experimental procedures with animals were in accordance with the guidelines of Institutional Animal Care and the French National Committee of Ethics. In addition, all experiments were conducted according to the Regulations for Animal Experimentation at the “Plateforme Animalerie” in the “Institut de Recherche en Santé de l’Université de Nantes (IRS-UN)” and approved by the French National Committee of Ethics.

## Author Contributions

PFC designed experiments and coordinated the project. MD, JB, AN, and PFC performed all experiments. GBC, FMV, JSF, SL and PFC interpreted and discussed the data. PFC wrote the manuscript. SL edited several versions of the manuscript. All authors have reviewed and approved the manuscript.

## Funding

This work was supported by funding from the LIGUE NATIONALE CONTRE LE CANCER, Comité InterRégional Grand Ouest, département de Loire Atlantique, d’Ille et Vilaine, Vendée et Côte d’Armorto PFC.

## Conflict of Interest Statement

The authors declare that the research was conducted in the absence of any commercial or financial relationships that could be construed as a potential conflict of interest.
