# Supplementary material for: Glyphosate Primes Mammary Cells for Tumorigenesis by Reprogramming the Epigenome in a TET3-Dependent Manner
Source: Front Genet. 2019 Sep 27;10:885. doi: 10.3389/fgene.2019.00885 (PMC6777643; doi:10.3389/fgene.2019.00885)
Supplement: Supplementary file 1 [file DataSheet_1.pdf]

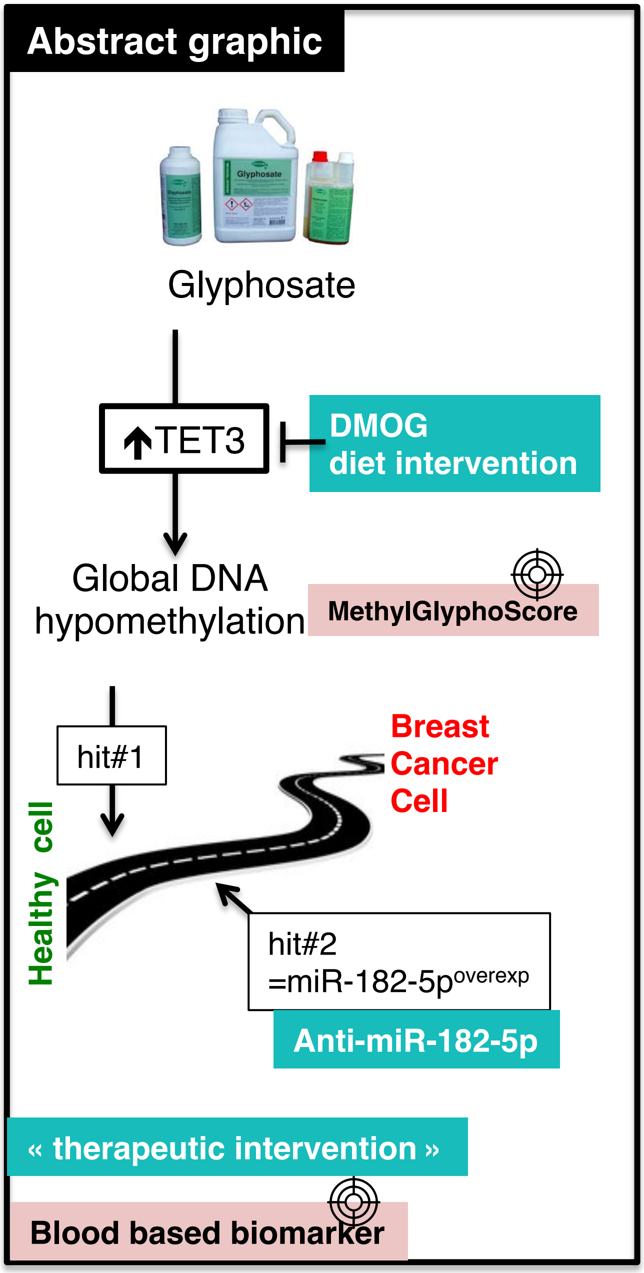

Abstract graphic
Glyphosate
TET3
DMOG
diet intervention
Global DNA
hypomethylation
MethylGlyphoScore
hit#1
Breast
Cancer
Cell
Healthy cell
hit#2
=miR-182-5p overexp
Anti-miR-182-5p
« therapeutic intervention »
Blood based biomarker

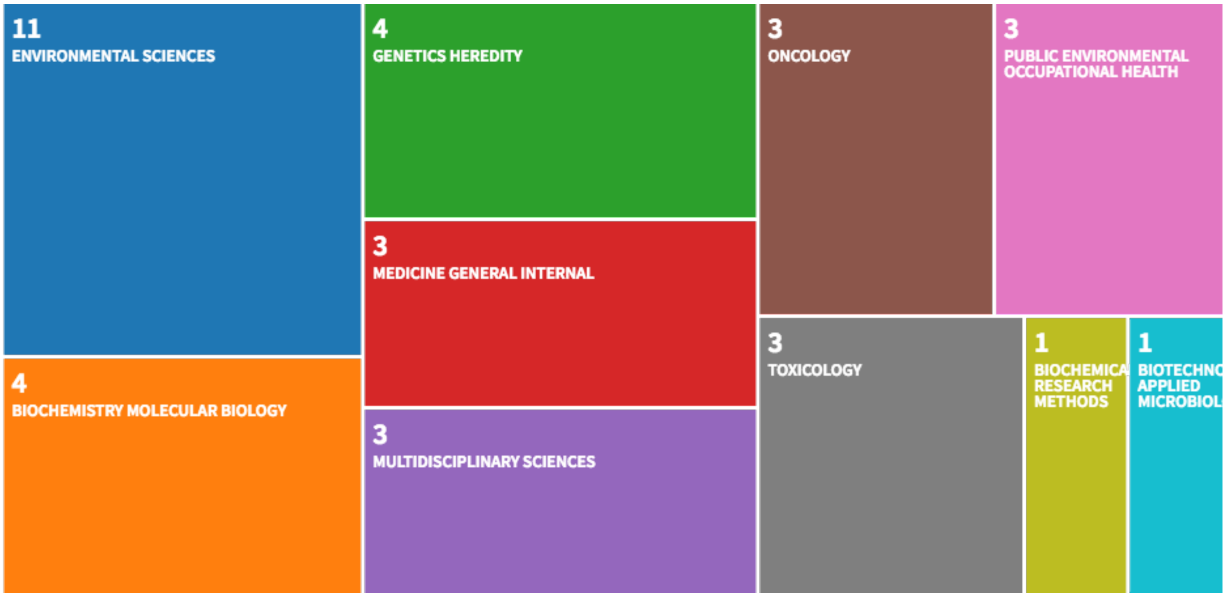

Figure S1. Illustration of the 26 records seen during the past five years, using combined key works “Pollutant AND epigenetic AND cancer risk” in the web of science.

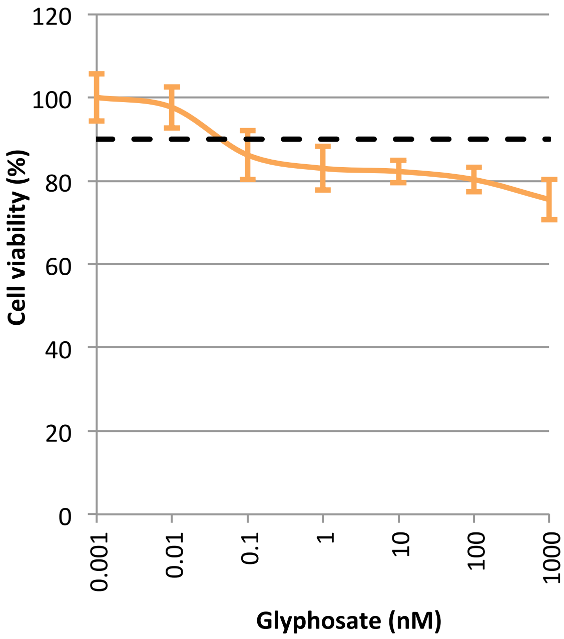

Figure S2. Percentage of viable MCF10A cells depending on glyphosate concentration.

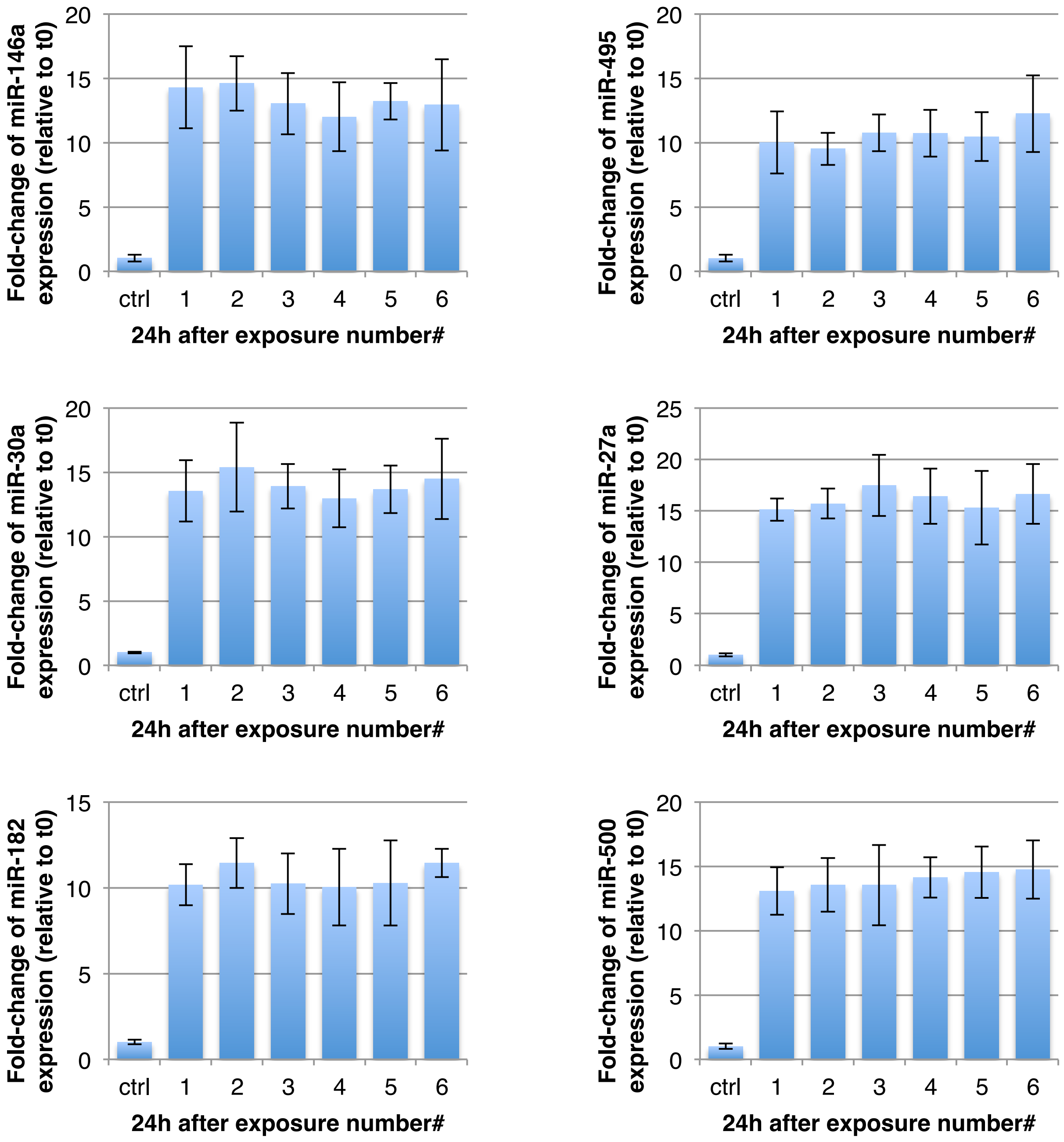

Figure S3. Efficacy of miRNA transfection analyzed by quantification of miRNA expression in MCF10A cells 24h after each transfection and exposure.to glyphosate (see treatment schedule Figure 2). Control (ctrl or t0) are nontransfected cells)

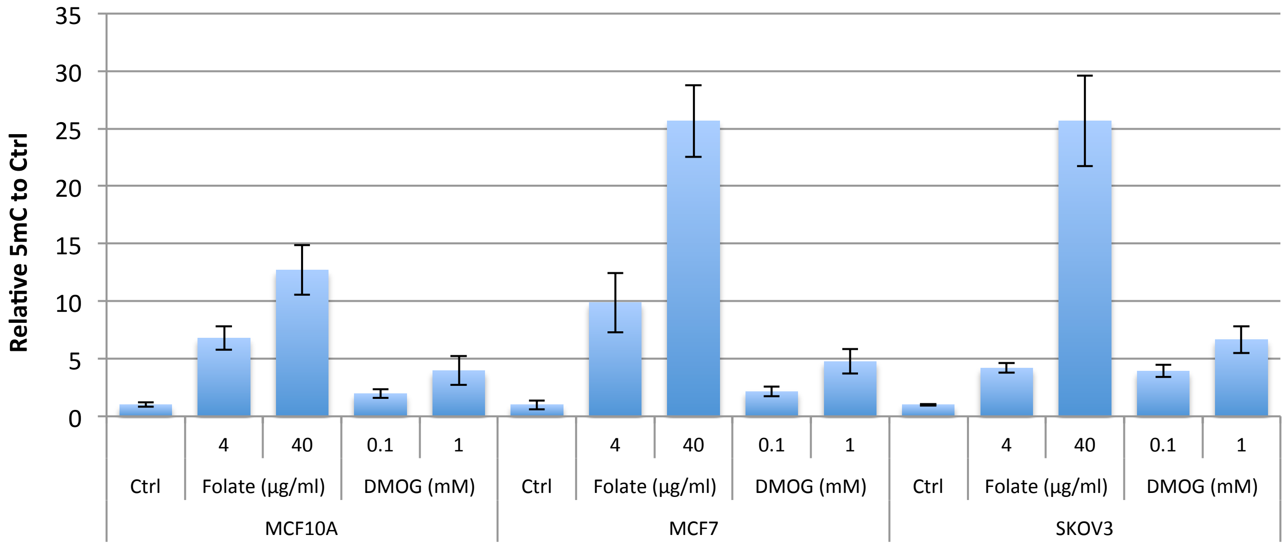

Figure S4. Efficiency of folate and DMOG to increase the global level of 5methylcytosine (5mC). 72h after Folate or DMOG addition, DNA was extrcated and ELISA was performed to estimate the 5mC level.

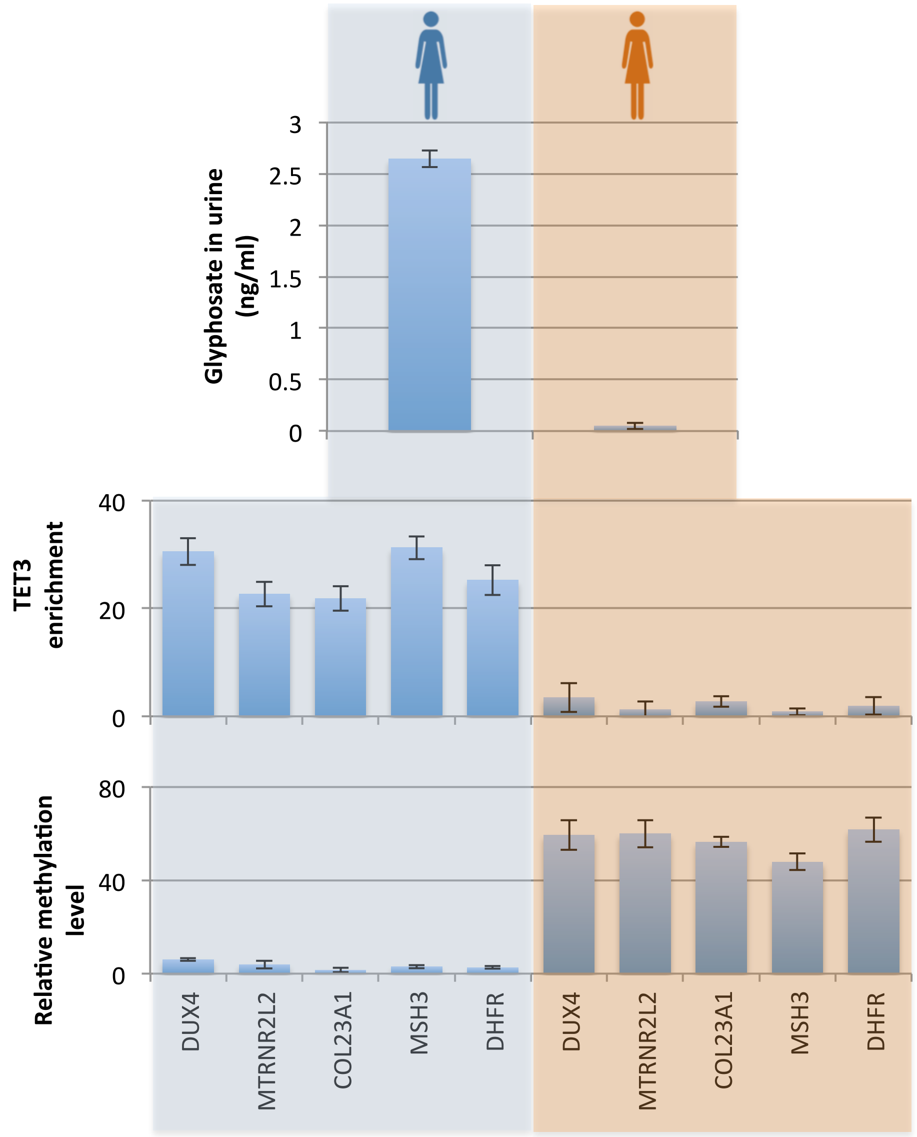

Figure S5. Status of TET3-related genes in breast tissues from women with either low or high glyphosate exposure.

Top graph: Glyphosate concentration in urine.

Middle graph: TET3 enrichment assessed via ChIP on *DUX4, MTRNR2L2, COL23A1, MSH3* and *DHFR genes.*

Bottom graph: Methylation levels of *DUX4, MTRNR2L2, COL23A1, MSH3* and *DHFR* relative to universal methylated DNA standard (Ozyme/Zymo, France).

The blue panels correspond to results from a woman with high exposure to glyphosate and the orange panels correspond to results from a women with low exposure to glyphosate.
