# Supplementary material for: Glyphosate Primes Mammary Cells for Tumorigenesis by Reprogramming the Epigenome in a TET3-Dependent Manner
Source: Front Genet. 2019 Sep 27;10:885. doi: 10.3389/fgene.2019.00885 (PMC6777643; doi:10.3389/fgene.2019.00885)
Supplement: Supplementary file 2 [file DataSheet_2.pdf]

Glyphosate primes mammary cells for tumorigenesis by reprogramming the epigenome in a TET3-dependent manner.

Manon Duforestel[1-2-3-4-5], Arulraj Nadaradjane[1-2-3-4-5], Gwenola Bougras-Cartron[1-2-3-4-5], Joséphine Briand[1-2-3-4-5], Christophe Olivier[1-2-6], Jean-Sébastien Frenel[1-2-3-4-5], François M Vallette[1-2-3-4-5], Sophie A. Lelièvre[7,8] and Pierre-François Cartron[1-2-3-4-5]

[1] CRCINA, INSERM, Université de Nantes, Nantes, France.

[2] Equipe Apoptose et Progression tumorale, LaBCT, Institut de Cancérologie de l'Ouest, Saint Herblain, France.

[3] Cancéropole Grand-Ouest, réseau Epigénétique (RepiCGO).

[4] EpiSAVMEN Consortium (Région Pays de la Loire).

[5] LabEX IGO, Université de Nantes, France.

[6] Service de toxicologie, Faculté de pharmacie de Nantes. Nantes, France

[7] Department of Basic Medical Sciences, Purdue University, West Lafayette, IN United States of America

[8] Purdue University Center for Cancer Research, West Lafayette, IN, United States of America

[*]Correspondence: pierre-francois.cartron@inserm.fr, CRCINA, INSERM U1232, Equipe Apoptose et Progression tumorale, LaBCT, Institut de Cancérologie de l'Ouest, Boulevard du Pr Jacques Monod, Saint Herblain, 44805, France.

Running title: Glyphosate-mediated breast cancer risk

Keywords: DNA methylation, TET, breast cancer, hypomethylation, epigenetic mark.

Supplementary File 1: **Kaplan-Meier illustrated the potential outcome of TET3 expression in panel of cancer according to KM plotter website.** KM plotter website (www.kmplot.com; In the meantime, please kindly cite our paper to support further development: Nagy Á, Lánczky A, Menyhárt O, Győrffy B: Validation of miRNA prognostic power in hepatocellular carcinoma using expression data of independent datasets, Scientific Reports, 2018;8:9277 | DOI:10.1038/s41598-018-27521-y) is a database having the ability to assess the effect of 54k genes on survival in a large number of cancer types (n=21).

Kaplan-Meier Plotter

Pan-cancer RNA-seq

Pan-cancer

KM plotter | Home | Download | Updates | Contact

The desired RNAseq ID is valid: TET3 (-),

**RNAseq ID:**	TET3	-
**Survival:**	OS	
**Auto select best cutoff:**	checked	
**Follow up threshold:**	all	
**Censore at threshold:**	checked	
**Compute median over entire database:**	false	
**Cutoff value used in analysis:**	1483	
**Expression range of the probe:**	131 - 5949	
**Invert HR values below 1:**	not checked	

Restrictions

Tumor type: Bladder Carcinoma

Restrictions

Stage:	all
Gender:	all
Race:	all
Grade:	all
Mutation burden:	all

Results

P value: 0.0999
FDR: 100%

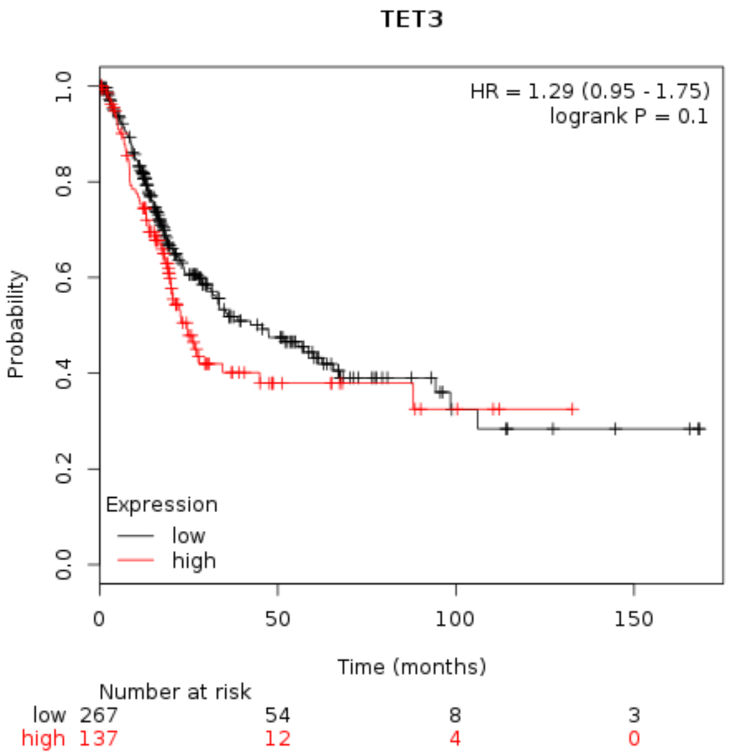

Download plot as a PDF

Download p values vs. cutoff table

**RNAseq ID:**	TET3	-
**Survival:**	OS	
**Auto select best cutoff:**	checked	
**Follow up threshold:**	all	
**Censore at threshold:**	checked	
**Compute median over entire database:**	false	
**Cutoff value used in analysis:**	781	
**Expression range of the probe:**	97 - 5141	
**Invert HR values below 1:**	not checked	

Restrictions

Tumor type: Breast cancer

Restrictions

Stage:	all
Gender:	all
Race:	all
Grade:	all
Mutation burden:	all

Results

P value: 0.0177
FDR: over 50%

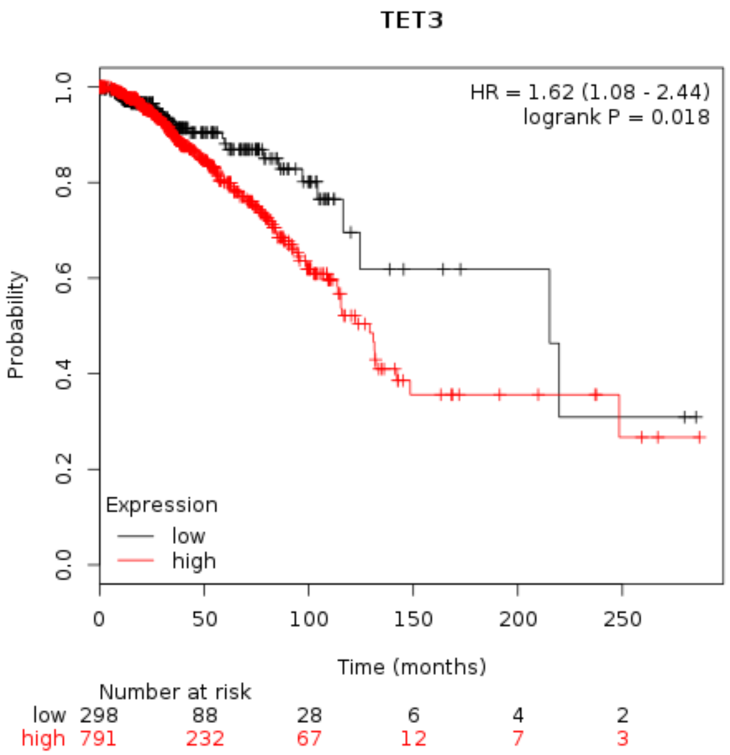

Download plot as a PDF

Download p values vs. cutoff table

**RNAseq ID:**	TET3 -
**Survival:**	OS
**Auto select best cutoff:**	checked
**Follow up threshold:**	all
**Censore at threshold:**	checked
**Compute median over entire database:**	false
**Cutoff value used in analysis:**	870
**Expression range of the probe:**	90 - 4627
**Invert HR values below 1:**	not checked

Restrictions

Tumor type: Cervical squamous cell carcinoma

Restrictions

Stage:	all
Gender:	all
Race:	all
Grade:	all
Mutation burden:	all

Results

P value: 0.016
FDR: over 50%

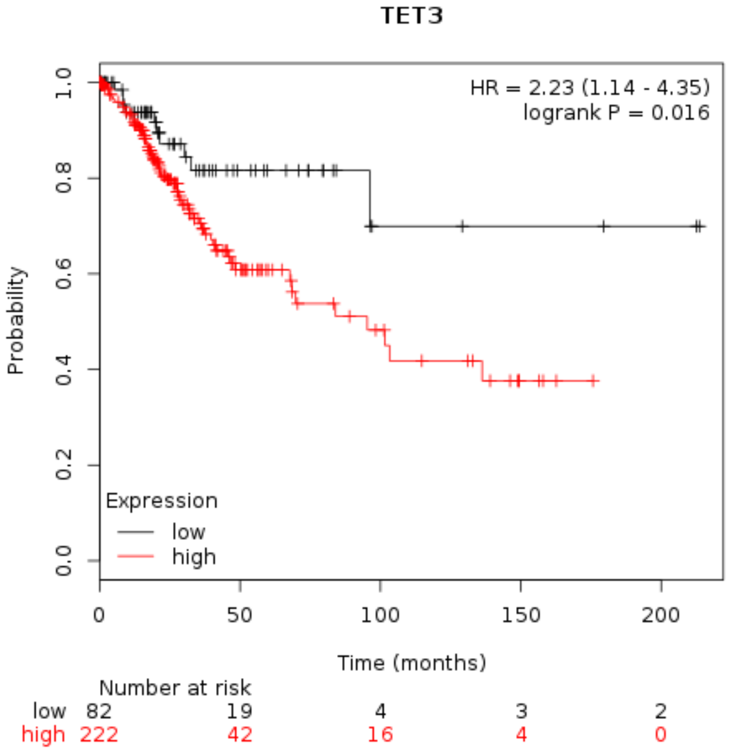

Download plot as a PDF

Download p values vs. cutoff table

**RNAseq ID:**	TET3 -
**Survival:**	OS
**Auto select best cutoff:**	checked
**Follow up threshold:**	all
**Censore at threshold:**	checked
**Compute median over entire database:**	false
**Cutoff value used in analysis:**	2318
**Expression range of the probe:**	621 - 7710
**Invert HR values below 1:**	not checked

Restrictions

Tumor type: Esophageal carcinoma

Restrictions

Stage:	all
Gender:	all
Race:	all
Grade:	all

Mutation burden: all

Results

P value: 0.1295
FDR: 100%

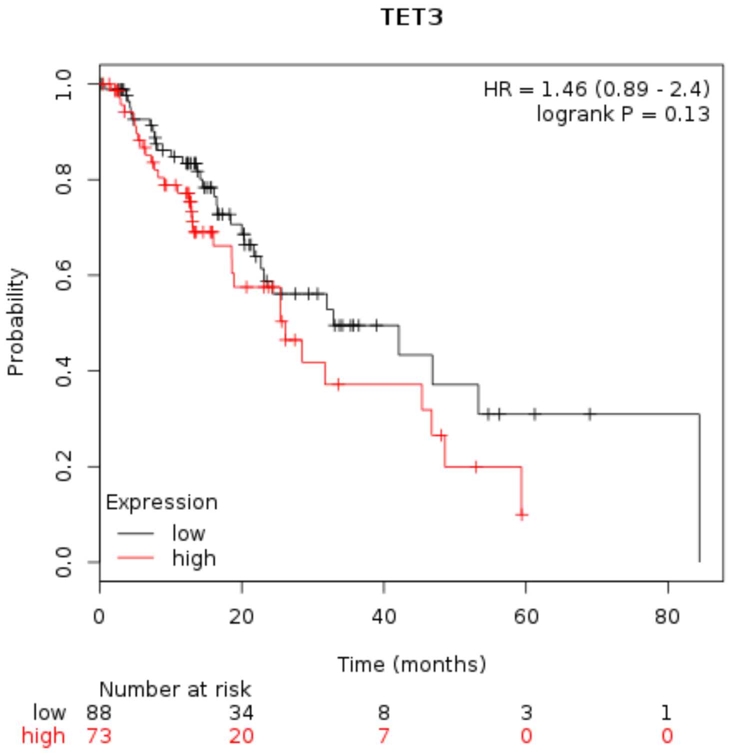

Download plot as a PDF

Download p values vs. cutoff table

**RNAseq ID:**	TET3 -
**Survival:**	OS
**Auto select best cutoff:**	checked
**Follow up threshold:**	all
**Censore at threshold:**	checked
**Compute median over entire database:**	false
**Cutoff value used in analysis:**	1245
**Expression range of the probe:**	95 - 7114
**Invert HR values below 1:**	not checked

Restrictions

Tumor type: Head-neck squamous cell carcinoma

Restrictions

Stage:	all
Gender:	all
Race:	all

Grade: all
Mutation burden: all

Results

P value: 0.0253
FDR: over 50%

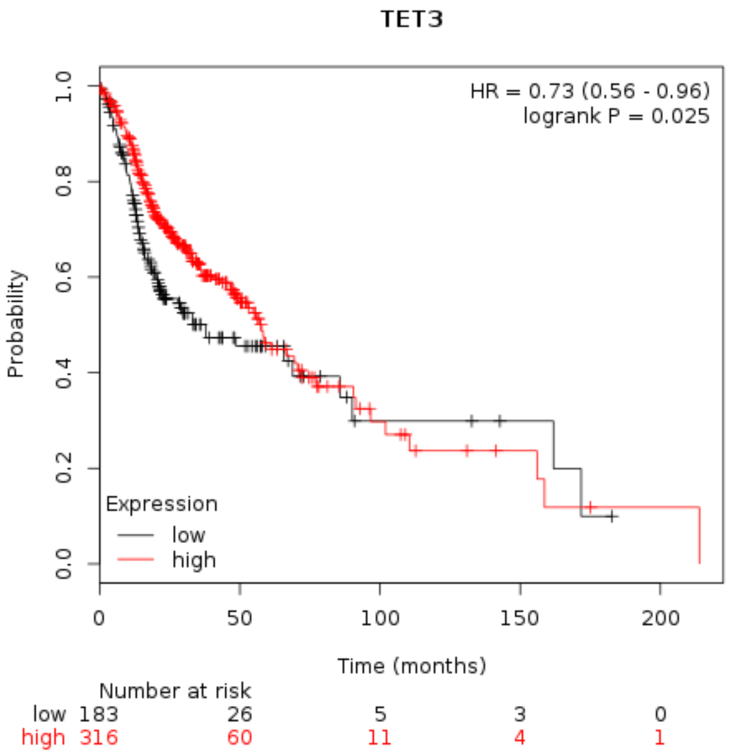

Download plot as a PDF

Download p values vs. cutoff table

**RNAseq ID:**	TET3 -
**Survival:**	OS
**Auto select best cutoff:**	checked
**Follow up threshold:**	all
**Censore at threshold:**	checked
**Compute median over entire database:**	false
**Cutoff value used in analysis:**	465
**Expression range of the probe:**	11 - 2270
**Invert HR values below 1:**	not checked

Restrictions

Tumor type: Kidney renal clear cell carcinoma

Restrictions

Stage: all

Gender: all
Race: all
Grade: all
Mutation burden: all

Results

P value: 0.0905
FDR: 100%

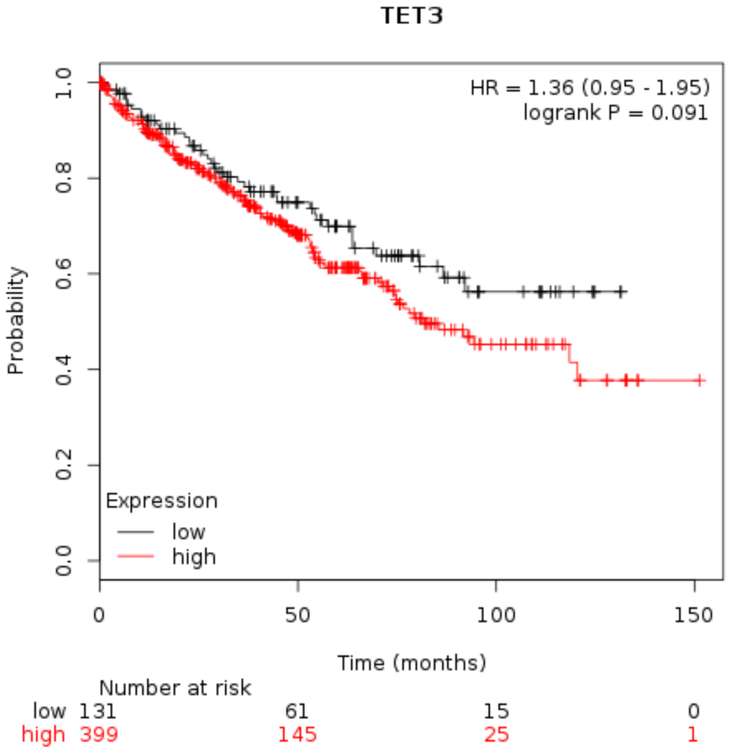

Download plot as a PDF

Download p values vs. cutoff table

**RNAseq ID:**	TET3 -
**Survival:**	OS
**Auto select best cutoff:**	checked
**Follow up threshold:**	all
**Censore at threshold:**	checked
**Compute median over entire database:**	false
**Cutoff value used in analysis:**	600
**Expression range of the probe:**	66 - 3118
**Invert HR values below 1:**	not checked

Restrictions

Tumor type: Kidney renal papillary cell carcinoma

Restrictions

Stage:	all
Gender:	all
Race:	all
Grade:	all
Mutation burden:	all

Results

P value: 0.0655
FDR: 100%

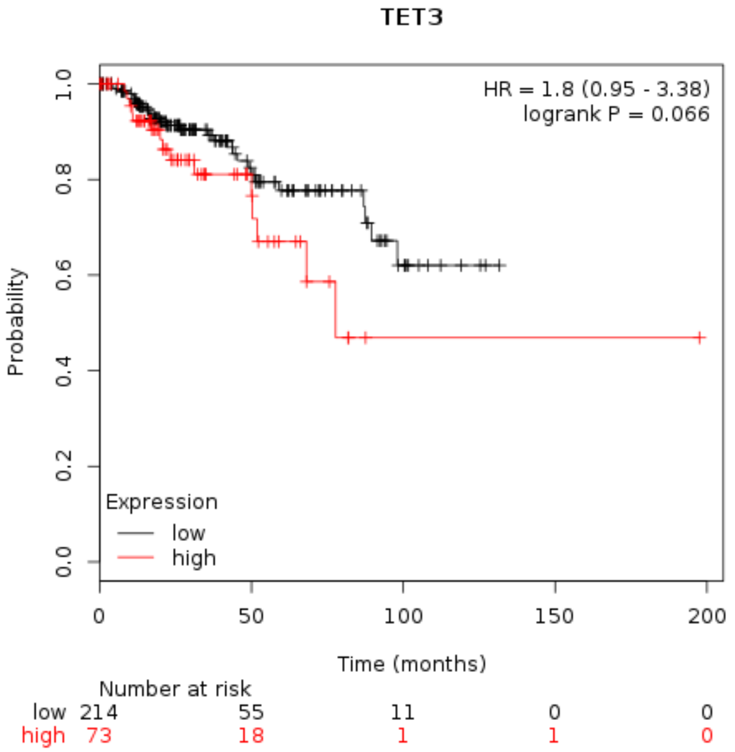

Download plot as a PDF

Download p values vs. cutoff table

**RNAseq ID:**	TET3	-
**Survival:**	OS	
**Auto select best cutoff:**	checked	
**Follow up threshold:**	all	
**Censore at threshold:**	checked	
**Compute median over entire database:**	false	
**Cutoff value used in analysis:**	359	
**Expression range of the probe:**	28 - 2145	
**Invert HR values below 1:**	not checked	

Restrictions

Tumor type: Liver hepatocellular carcinoma

Restrictions

Stage:	all
Gender:	all
Race:	all
Grade:	all
Mutation burden:	all

Results

P value: 0.0014
FDR: 20%

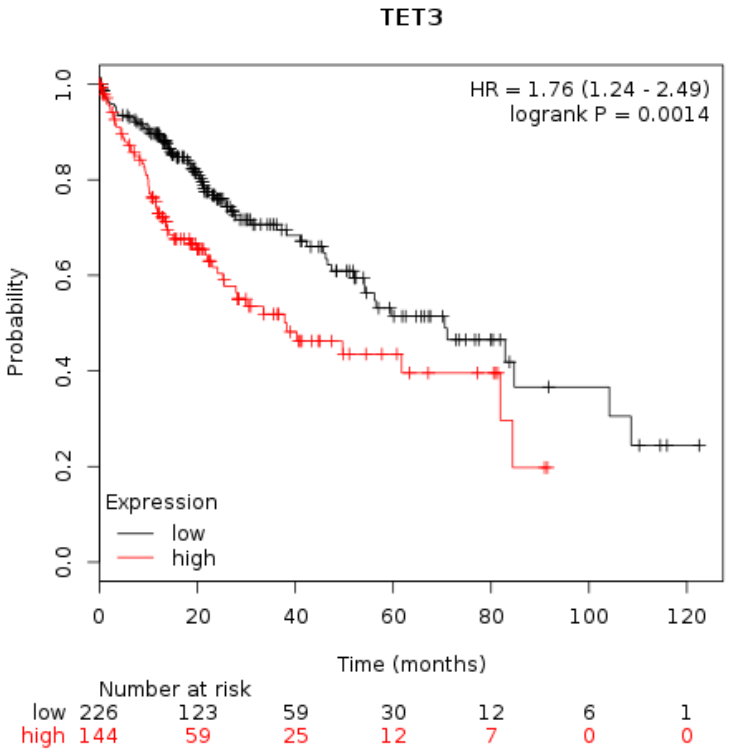

Download plot as a PDF

Download p values vs. cutoff table

**RNAseq ID:**	TET3 -
**Survival:**	OS
**Auto select best cutoff:**	checked
**Follow up threshold:**	all
**Censore at threshold:**	checked
**Compute median over entire database:**	false
**Cutoff value used in analysis:**	777
**Expression range of the probe:**	130 - 4045
**Invert HR values below 1:**	not checked

Restrictions

Tumor type: Lung adenocarcinoma

Restrictions

Stage:	all
Gender:	all
Race:	all
Grade:	all
Mutation burden:	all

Results

P value: 0.0627
FDR: 100%

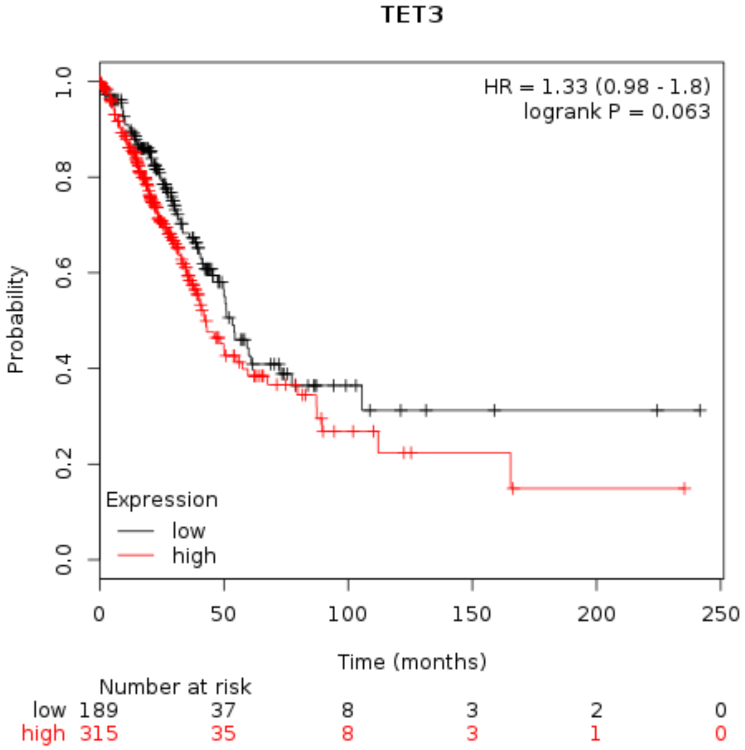

Download plot as a PDF

Download p values vs. cutoff table

**RNAseq ID:**	TET3	-
**Survival:**	OS	
**Auto select best cutoff:**	checked	
**Follow up threshold:**	all	
**Censore at threshold:**	checked	
**Compute median over entire database:**	false	
**Cutoff value used in analysis:**	2587	
**Expression range of the probe:**	146 - 6967	
**Invert HR values below 1:**	not checked	

Restrictions

Tumor type: Lung squamous cell carcinoma

Restrictions

Stage:	all
Gender:	all
Race:	all
Grade:	all
Mutation burden:	all

Results

P value: 0.0659
FDR: 100%

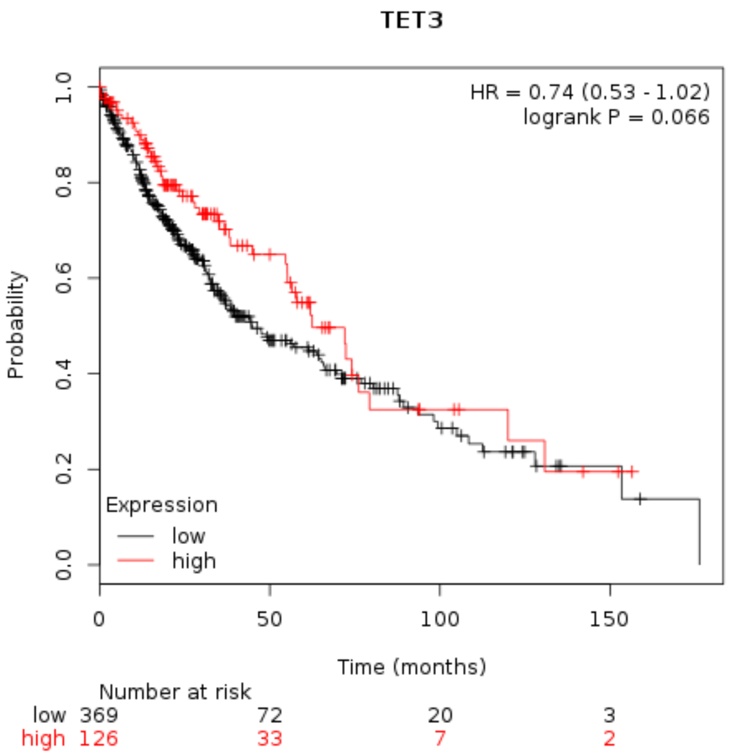

Download plot as a PDF

Download p values vs. cutoff table

**RNAseq ID:**	TET3 -
**Survival:**	OS
**Auto select best cutoff:**	checked
**Follow up threshold:**	all
**Censore at threshold:**	checked
**Compute median over entire database:**	false
**Cutoff value used in analysis:**	791
**Expression range of the probe:**	137 - 4458

Invert HR values below 1: not checked

Restrictions

Tumor type: Ovarian cancer

Restrictions

Stage:	all
Gender:	all
Race:	all
Grade:	all
Mutation burden:	all

Results

P value: 0.0801
FDR: 100%

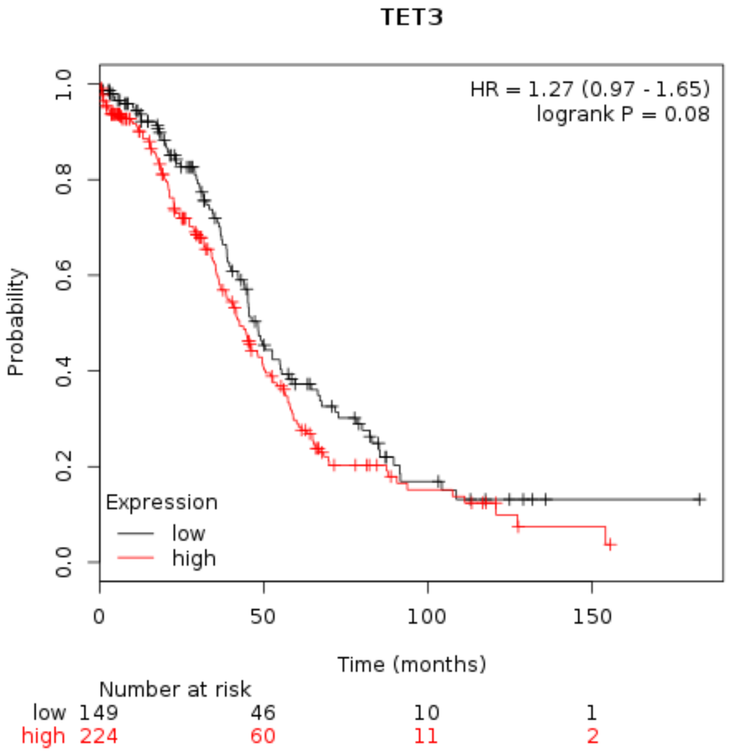

Download plot as a PDF

Download p values vs. cutoff table

**RNAseq ID:**	TET3 -
**Survival:**	OS
**Auto select best cutoff:**	checked
**Follow up threshold:**	all
**Censore at threshold:**	checked
**Compute median over entire database:**	false
**Cutoff value used in analysis:**	849

**Expression range of the probe:**	74 - 1303
**Invert HR values below 1:**	not checked

Restrictions

Tumor type: Pancreatic ductal adenocarcinoma

Restrictions

Stage:	all
Gender:	all
Race:	all
Grade:	all
Mutation burden:	all

Results

P value: 0.1045
FDR: 100%

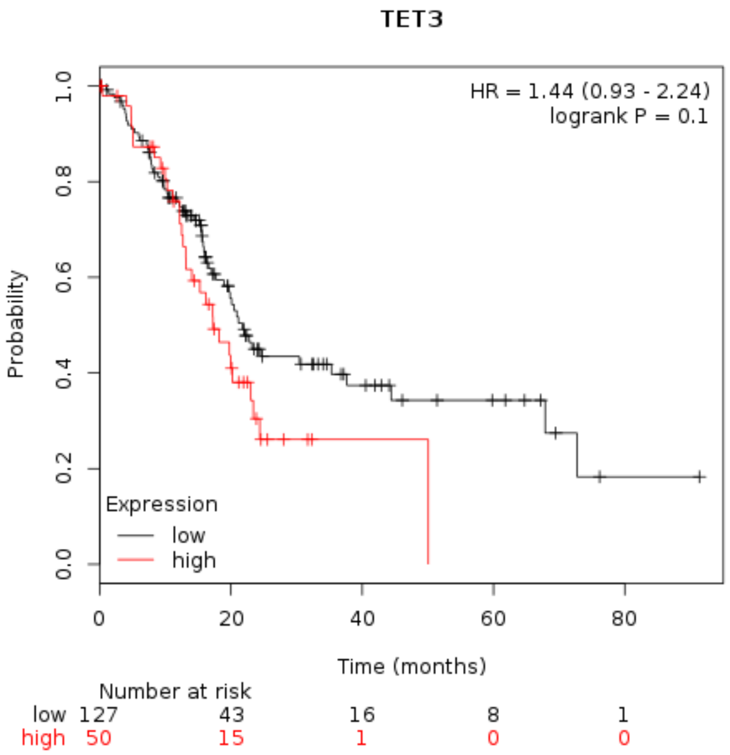

Download plot as a PDF

Download p values vs. cutoff table

**RNAseq ID:**	TET3 -
**Survival:**	OS
**Auto select best cutoff:**	checked
**Follow up threshold:**	all
**Censore at threshold:**	checked

Compute median over entire database: false
Cutoff value used in analysis: 302
Expression range of the probe: 42 - 1229
Invert HR values below 1: not checked

Restrictions

Tumor type: Pheochromocytoma and Paraganglioma

Restrictions

Stage:	all
Gender:	all
Race:	all
Grade:	all
Mutation burden:	all

Results

P value: 0.0088
FDR: over 50%

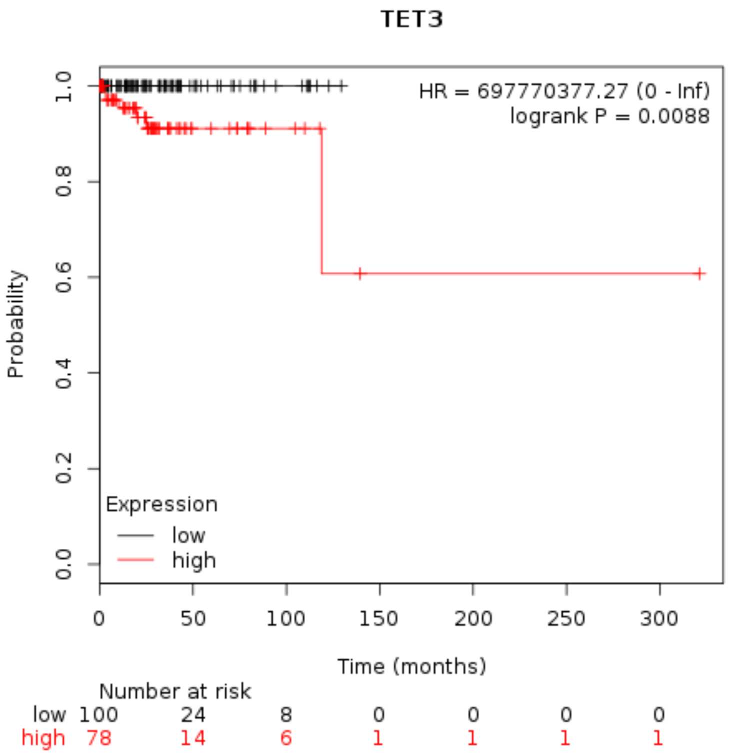

Download plot as a PDF

Download p values vs. cutoff table

RNAseq ID: TET3 -
Survival: OS
Auto select best cutoff: checked
Follow up threshold: all

**Censore at threshold:**	checked
**Compute median over entire database:**	false
**Cutoff value used in analysis:**	1286
**Expression range of the probe:**	150 - 2312
**Invert HR values below 1:**	not checked

Restrictions

Tumor type: Rectum adenocarcinoma

Restrictions

Stage:	all
Gender:	all
Race:	all
Grade:	all
Mutation burden:	all

Results

P value: 0.062
FDR: 100%

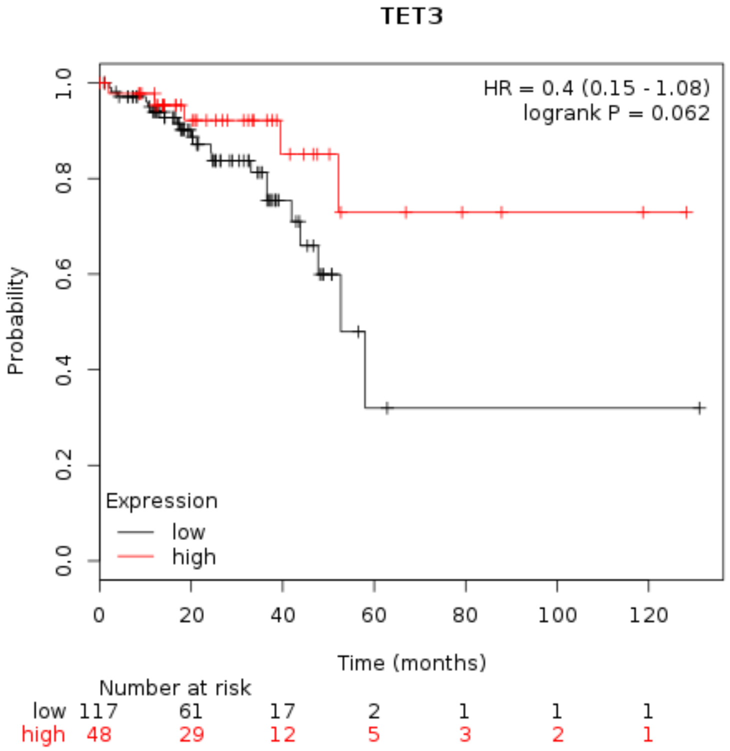

Download plot as a PDF

Download p values vs. cutoff table

**RNAseq ID:**	TET3	-
**Survival:**	OS	

**Auto select best cutoff:**	checked
**Follow up threshold:**	all
**Censore at threshold:**	checked
**Compute median over entire database:**	false
**Cutoff value used in analysis:**	450
**Expression range of the probe:**	45 - 3781
**Invert HR values below 1:**	not checked

Restrictions

Tumor type: Sarcoma

Restrictions

Stage:	all
Gender:	all
Race:	all
Grade:	all
Mutation burden:	all

Results

P value: 0.0505
FDR: 100%

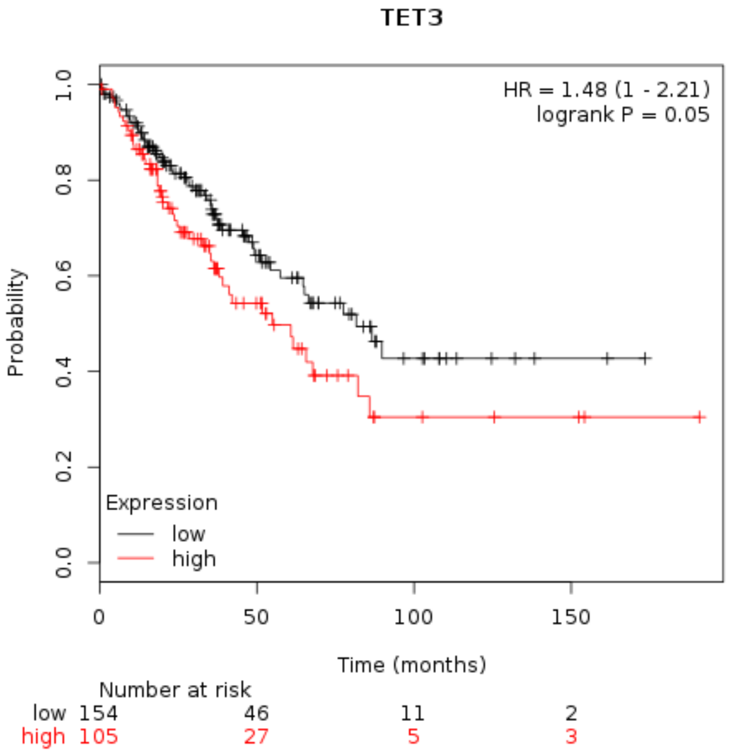

Download plot as a PDF

Download p values vs. cutoff table

RNAseq ID: TET3 -

**Survival:**	OS
**Auto select best cutoff:**	checked
**Follow up threshold:**	all
**Censore at threshold:**	checked
**Compute median over entire database:**	false
**Cutoff value used in analysis:**	1012
**Expression range of the probe:**	273 - 4358
**Invert HR values below 1:**	not checked

Restrictions

Tumor type: Stomach adenocarcinoma

Restrictions

Stage:	all
Gender:	all
Race:	all
Grade:	all
Mutation burden:	all

Results

P value: 0.199
FDR: 100%

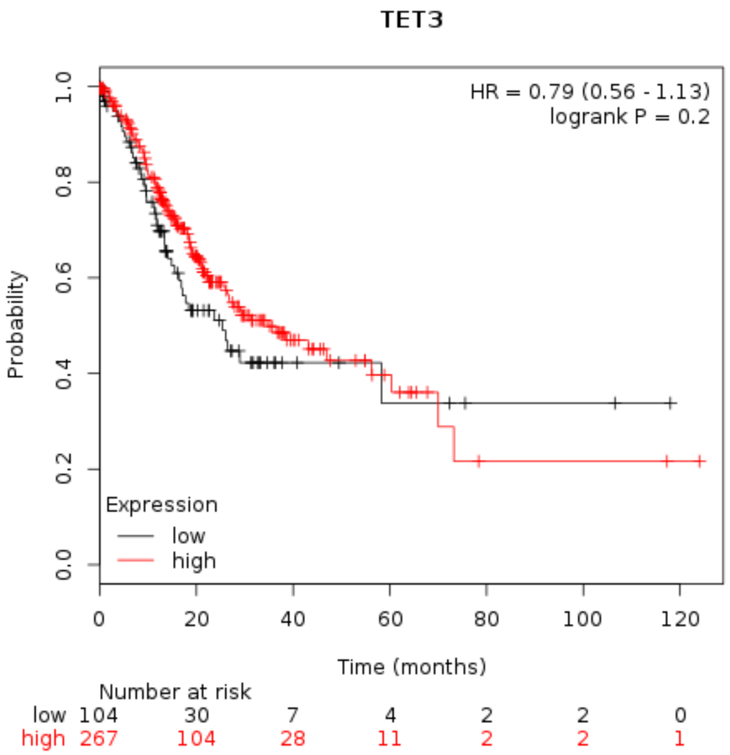

Download plot as a PDF

Download p values vs. cutoff table

**RNAseq ID:**	TET3 -
**Survival:**	OS
**Auto select best cutoff:**	checked
**Follow up threshold:**	all
**Censore at threshold:**	checked
**Compute median over entire database:**	false
**Cutoff value used in analysis:**	579
**Expression range of the probe:**	101 - 3565
**Invert HR values below 1:**	not checked

Restrictions

Tumor type: Testicular Germ Cell Tumor

Restrictions

Stage:	all
Gender:	all
Race:	all
Grade:	all
Mutation burden:	all

Results

P value: 0.5297
FDR: 100%

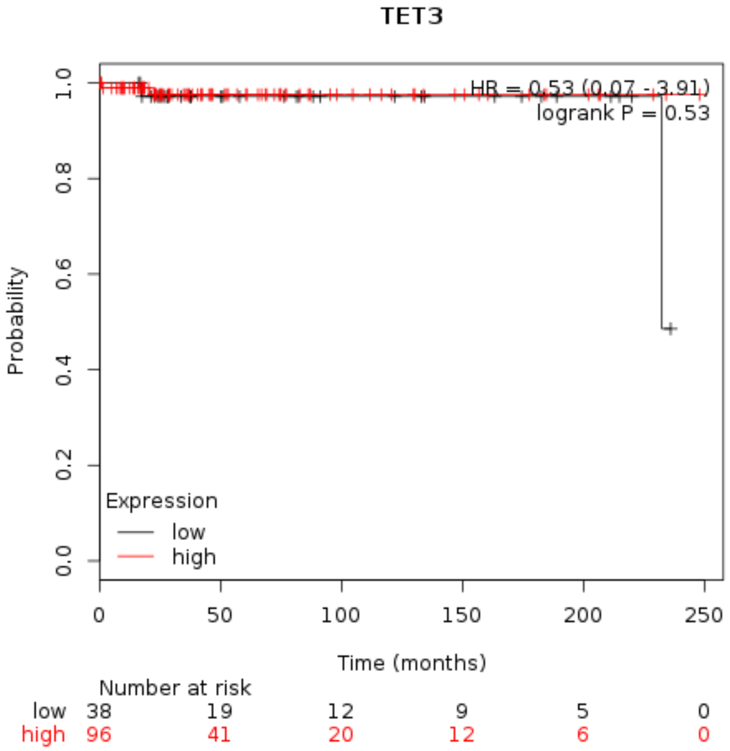

Download plot as a PDF

Download p values vs. cutoff table

**RNAseq ID:**	TET3 -
**Survival:**	OS
**Auto select best cutoff:**	checked
**Follow up threshold:**	all
**Censore at threshold:**	checked
**Compute median over entire database:**	false
**Cutoff value used in analysis:**	786
**Expression range of the probe:**	49 - 1907
**Invert HR values below 1:**	not checked

Restrictions

Tumor type: Thymoma

Restrictions

Stage:	all
Gender:	all
Race:	all
Grade:	all
Mutation burden:	all

Results

P value: 0.0441
FDR: over 50%

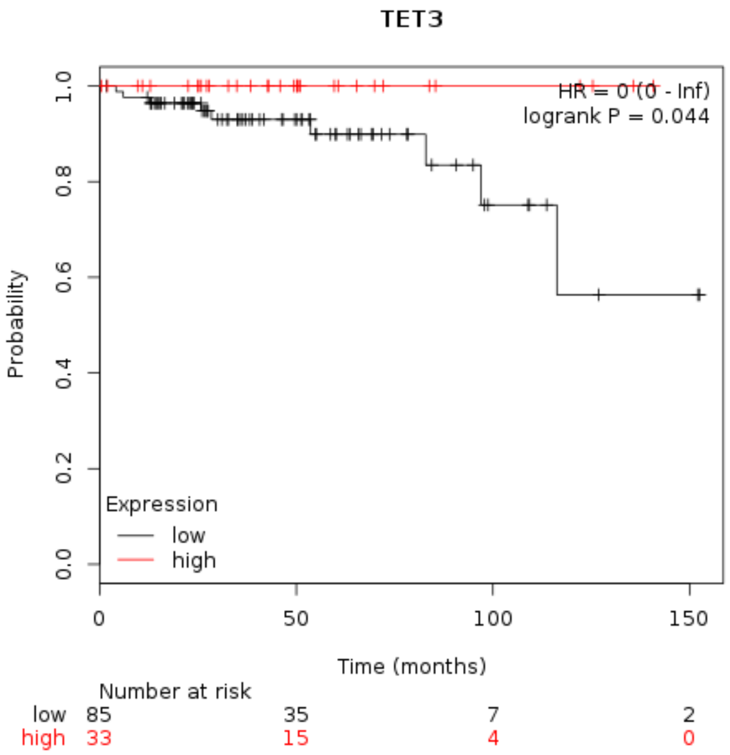

Download plot as a PDF

Download p values vs. cutoff table

**RNAseq ID:**	TET3	-
**Survival:**	OS	
**Auto select best cutoff:**	checked	
**Follow up threshold:**	all	
**Censore at threshold:**	checked	
**Compute median over entire database:**	false	
**Cutoff value used in analysis:**	602	
**Expression range of the probe:**	109 - 1553	
**Invert HR values below 1:**	not checked	

Restrictions

Tumor type: Thyroid carcinoma

Restrictions

Stage:	all
Gender:	all
Race:	all
Grade:	all
Mutation burden:	all

Results

**P value:**	0.0157
**FDR:**	over 50%

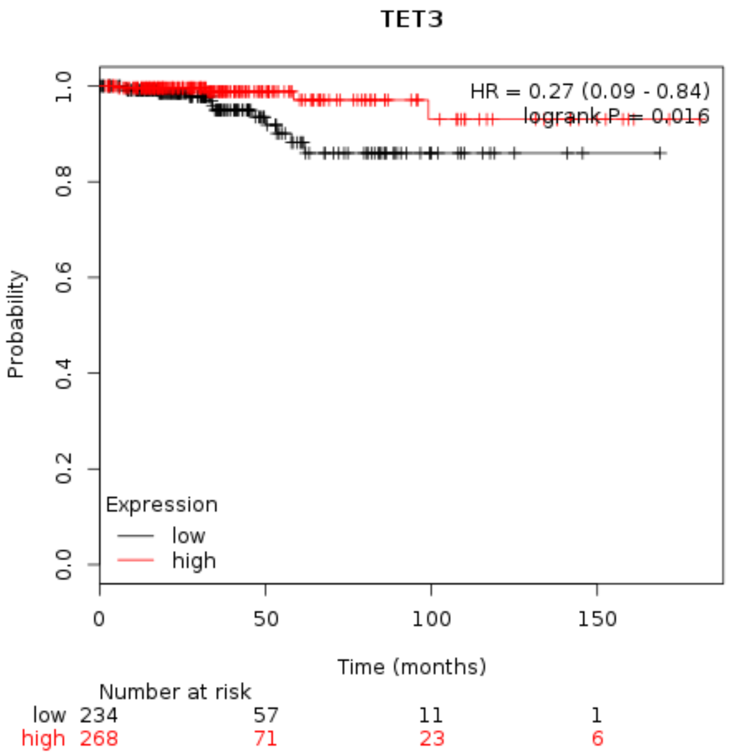

Download plot as a PDF

Download p values vs. cutoff table

**RNAseq ID:**	TET3 -
**Survival:**	OS
**Auto select best cutoff:**	checked
**Follow up threshold:**	all
**Censore at threshold:**	checked
**Compute median over entire database:**	false
**Cutoff value used in analysis:**	1279
**Expression range of the probe:**	77 - 4774
**Invert HR values below 1:**	not checked

Restrictions

Tumor type: Uterine corpus endometrial carcinoma

Restrictions

Stage:	all
Gender:	all
Race:	all
Grade:	all
Mutation burden:	all

Results

P value: 0.0094
FDR: over 50%

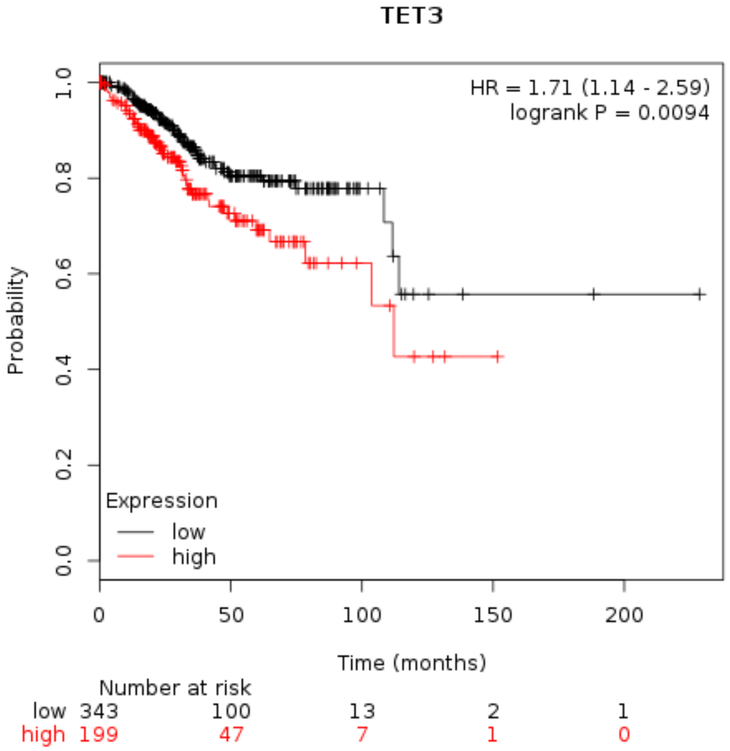

Download plot as a PDF

Download p values vs. cutoff table

You can save the plots by right-clicking the image and then selecting "Save image as...". To generate a high resolution TIFF image, please adjust the "Settings" in the analysis page.

KMplot.com Copyright © 2009-2018 | Contact
