# Supplementary material for: Glyphosate Primes Mammary Cells for Tumorigenesis by Reprogramming the Epigenome in a TET3-Dependent Manner
Source: Front Genet. 2019 Sep 27;10:885. doi: 10.3389/fgene.2019.00885 (PMC6777643; doi:10.3389/fgene.2019.00885)
Supplement: Supplementary file 3 [file DataSheet_3.pdf]

# Glyphosate primes mammary cells for tumorigenesis by reprogramming the epigenome in a TET3-dependent manner.

Manon Duforestel[1-2-3-4-5], Arulraj Nadaradjane[1-2-3-4-5], Gwenola Bougras-Cartron[1-2-3-4-5], Joséphine Briand[1-2-3-4-5], Christophe Olivier[1-2-6], Jean-Sébastien Frenel[1-2-3-4-5], François M Vallette[1-2-3-4-5], Sophie A. Lelièvre[7,8] and Pierre-François Cartron[1-2-3-4-5]

[1] CRCINA, INSERM, Université de Nantes, Nantes, France.

[2] Equipe Apoptose et Progression tumorale, LaBCT, Institut de Cancérologie de l'Ouest, Saint Herblain, France.

[3] Cancéropole Grand-Ouest, réseau Epigénétique (RepiCGO).

[4] EpiSAVMEN Consortium (Région Pays de la Loire).

[5] LabEX IGO, Université de Nantes, France.

[6] Service de toxicologie, Faculté de pharmacie de Nantes. Nantes, France

[7] Department of Basic Medical Sciences, Purdue University, West Lafayette, IN United States of America

[8] Purdue University Center for Cancer Research, West Lafayette, IN, United States of America

[*]Correspondence: pierre-francois.cartron@inserm.fr, CRCINA, INSERM U1232, Equipe Apoptose et Progression tumorale, LaBCT, Institut de Cancérologie de l'Ouest, Boulevard du Pr Jacques Monod, Saint Herblain, 44805, France.

**Running title:** Glyphosate-mediated breast cancer risk

**Keywords:** DNA methylation, TET, breast cancer, hypomethylation, epigenetic mark.

**Supplementary File 2**: **Kaplan-Meier illustrated the potential outcome of DUX4 expression in Breast cancer according to** BC-GenExMiner and **KM plotter website.**

BC-GenExMiner (http://bcgenex.centregauducheau.fr) is a statistical mining tool of published *annotated* breast cancer transcriptomic data (*DNA microarrays* [n = 10 012] and *RNA-seq* [n = 4 713]. Here, we have used BC-GenExMiner to draw the Kaplan-Meier curves for DUX4 in Breast cancer.

KM plotter website (www.kmplot.com; In the meantime, please kindly cite our paper to support further development: Nagy Á, Lánczky A, Menyhárt O, Győrffy B: Validation of miRNA prognostic power in hepatocellular carcinoma using expression data of independent datasets, Scientific Reports, 2018;8:9277 | DOI:10.1038/s41598-018-27521-y) is a database having the ability to assess the effect of 54k genes on survival in a large number of cancer types (n=21). Here, we have used KM plotter to draw the Kaplan-Meier curves for DUX4 in Breast cancer.

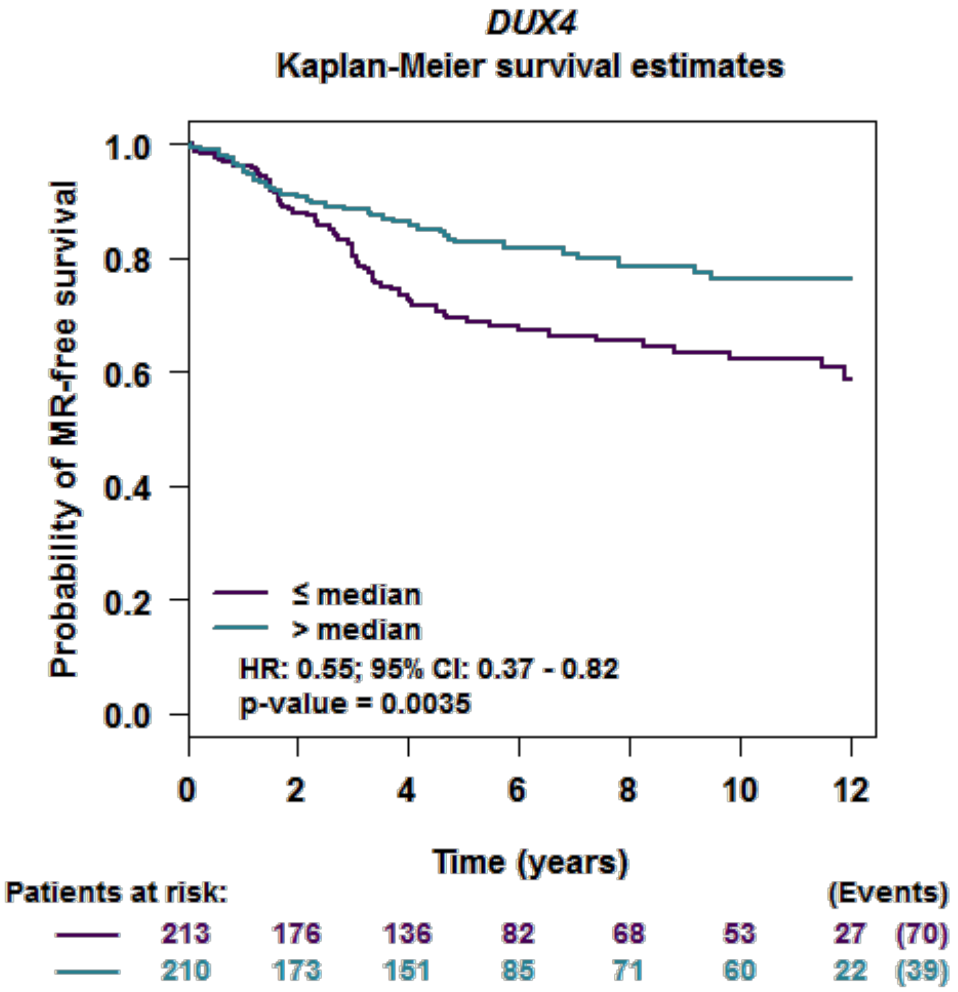
*DUX4*
Kaplan-Meier survival estimates
Probability of MR-free survival
1.0
0.8
0.6
0.4
0.2
0.0
≤ median
> median
HR: 0.55; 95% CI: 0.37 - 0.82
p-value = 0.0035
0
2
4
6
8
10
12
Time (years)
Patients at risk:
(Events)
213 176 136 82 68 53 27 (70)
210 173 151 85 71 60 22 (39)

Kaplan-Meier Plotter

Breast Cancer

Breast Cancer

KM plotter | Home | Download | Updates | Contact

The desired is valid: 208201_at (LOC653544),

| | | |
|---|---|---|
| **Affy ID:** | 208201_at | LOC653544, LOC653545, DUX3, ... |
| **Survival:** | RFS | |
| **Split patients by:** | median | |
| **Follow up threshold:** | all | |
| **Censore at threshold:** | checked | |
| **Compute median over entire database:** | false | |
| **Cutoff value used in analysis:** | 17 | |
| **Expression range of the probe:** | 1 - 667 | |
| **Probe set option:** | user selected probe set | |
| **Invert HR values below 1:** | not checked | |

## Restrictions

| | |
|---|---|
| ER status: | all |
| *derive ER status from gene expression data:* | not checked |
| PR status: | all |
| HER2 status: | all |
| Intrinsic subtype: | all |
| Lymph node status: | all |
| Grade: | all |
| TP53 status: | all |
| Pietenpol subtype: | all |
| Use earlier release of the database: | all |
| Use following dataset for the analysis: | all |

## Quality control

| | |
|---|---|
| Remove redundant samples: | checked |
| Array quality control: | exclude biased arrays |
| Proportional hazards assumption: | 0 |

## Cohort

Cohorts: not selected

## Results

**P value:** 0.0166

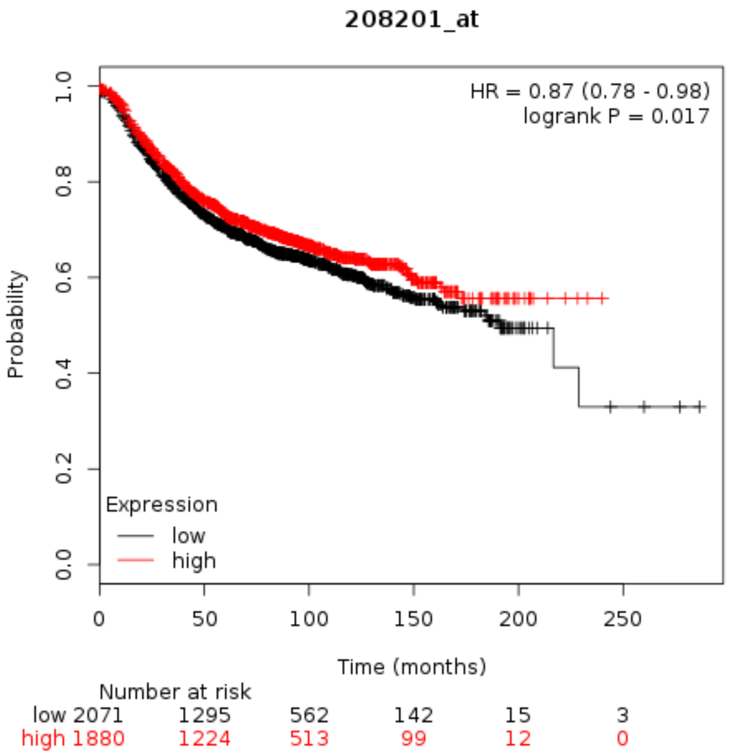


Download plot as a PDF

---

You can save the plots by right-clicking the image and then selecting "Save image as...". To generate a high resolution TIFF image, please adjust the "Settings" in the analysis page.

KMplot.com Copyright © 2009-2019 | Contact
